# Atomistic weak interaction criterion for the specificity of liquid metal embrittlement

**DOI:** 10.1038/s41598-022-10593-2

**Published:** 2022-07-04

**Authors:** Masatake Yamaguchi, Tomohito Tsuru, Mitsuhiro Itakura, Eiji Abe

**Affiliations:** 1 Center for Computational Science & e-Systems, Japan Atomic Energy Agency, 2-4 Shirakata, Tokai-mura, Naka-gun, Ibaraki, 319-1195 Japan; 2 Department of Materials Science and Engineering, The University of Tokyo, 7-3-1 Hongo, Bunkyo-ku, Tokyo, 113-8656 Japan; 3 Elements Strategy Initiative for Structural Materials, Kyoto University, Yoshida-honmachi, Sakyo-ku, Kyoto, 606-8501 Japan; 4 Nuclear Science and Engineering Center, Japan Atomic Energy Agency, 2-4 Shirakata, Tokai-mura, Naka-gun, Ibaraki, 319-1195 Japan; 5 Center for Computational Science & e-Systems, Japan Atomic Energy Agency, 178-4 Wakashiba, Kashiwa, Chiba 277-0871 Japan; 6 Research Center for Structural Materials, National Institute of Materials Science, 1-2-1 Sengen, Tsukuba, Ibaraki 305-0047 Japan

**Keywords:** Metals and alloys, Atomistic models

## Abstract

Liquid metal embrittlement (LME) occurs in some solid–liquid metal elements’ couples (e.g., Fe-Zn and Al-Ga), called specificity. Although some material parameters like solubility and bonding energy were suggested as controlling factors, none could be attributed satisfactorily. Here we have unveiled the primary factor that governs the specificity of LME. From first-principles calculations compared with a systematic surveillance test result, we found that the grain-boundary (GB) adsorption energy shows near-zero values in all embrittling couples; the interaction between solid and liquid metal atoms is weak when an atom from the liquid state penetrates the grain boundary of the solid. Furthermore, we found that the calculated surface adsorption energy that promotes bond-breaking does not correlate to the specificity. Therefore, we consider that the penetration of a liquid metal atom surrounded by weakly interacting solid metal atoms is necessary before the bond-breaking assisted by surface adsorption occurs at a microcrack tip. This mechanism is also applicable for transgranular cracking along low-energy boundaries and crystal planes. While liquid metal atoms penetrate and diffuse into solid GB macroscopically before cracking, liquid metal’s surface adsorption stronger than GB adsorption should promote the bond-breaking of solid metal. In conclusion, the atomistic penetration precedes the surface-adsorption-assisted bond-breaking and controls the specificity of LME.

## Introduction

Solid metals are susceptible to brittle fracture when they come in contact with certain liquid metals (e.g., Fe–Zn and Al–Ga). This phenomenon is called liquid metal embrittlement, discovered in 1874^1^. One of the most severe embrittling cases is polycrystalline aluminum alloys embrittled by liquid gallium; liquid gallium penetrates aluminum GBs macroscopically at high speed^2^. Zinc atoms’ penetration, diffusion, and subsequent liquid zinc penetration into steel GBs have been observed because molten zinc embrittlement has attracted much attention by increasing steels’ strength^3,4^, especially in the automotive industry. Luo et al. have clarified that bismuth forms bilayer interfacial phase along nickel GBs^5^. In these cases, the subsequent embrittlement occurs due to the change in GB strength induced by liquid metal atoms existing in the GB before cracking.

On the other hand, embrittlement occurs even when exposed to the liquid metal without detectable atomic diffusion or liquid penetration into GBs, lath boundaries in martensitic steel, and crystal lattice planes. Although liquid mercury atoms do not seem to penetrate and diffuse into high-strength steel’s GBs and lath boundaries, embrittlement does occur along those boundaries^6^. Even single crystal aluminum exhibits embrittlement by liquid gallium^7^ after significant plastic deformation. The extremest example is the mercury embrittlement of single and polycrystal zinc; fracture occurs within seconds after coating mercury on stress-loaded zinc^8^. In this way, LME occurs even if liquid metal atoms do not exist along the cracking path before fracture.

One of the curious phenomena that many researchers have tried to solve is why embrittlement occurs or does not occur depending on a given solid–liquid metal elements’ couple under a specific test condition. Such selectivity is called the specificity of LME^9^, which has been known for over a century, yet it is not well understood. Some material parameters suggested as controlling factors are solubility, interatomic bonding energy, and intermetallics formation. However, none of them could be attributed to the specificity of LME satisfactorily^10,11^. We considered that the crucial atomistic mechanism of LME must be hidden behind the mystery of the LME specificity.

**Figure 1:**
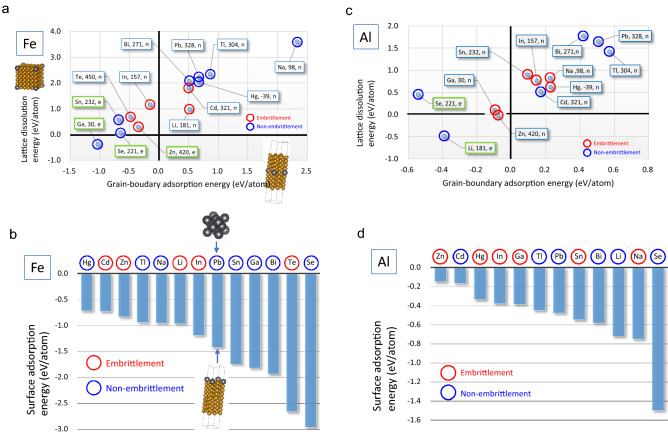
Calculated atomistic energies of liquid metal elements compared with the specificity of LME. (**a**) GB adsorption energies on FeΣ3(111) symmetrical tilt GB and lattice dissolution energies in Fe of liquid metal elements. (**b**) Surface adsorption energies of liquid metal elements on Fe(111) surface, which is a fracture surface of FeΣ3(111) GB. Inserted figures show computational cells of the Fe-Pb case. (**c**) GB adsorption energies on AlΣ5(012) symmetrical tilt GB and lattice dissolution energies in Al of liquid metal elements. (**d**) Surface adsorption energies of liquid metal elements on Al(012) surface, which is a fracture surface of AlΣ5(012) GB. Negative energy indicates attractive interaction between atoms. Embrittlement or Non-embrittlement refers to Rostoker et al.’s specificity data of LME^10^, as shown in Table 1. The number in the label box is the melting temperature (degrees of Celsius). The green box indicates that a binary intermetallic compound exists (**e**), whereas the other box does not (n)^22^.

Herein, for the first time, we have realized the primary factor that governs the specificity of LME. From first-principles calculations, we found an excellent correlation between our calculated GB adsorption energy of a liquid metal atom in solid metal and the specificity of LME, as shown in Fig. 1a–c. Compared with the systematic surveillance test result (Table 1) by Rostoker et al.^10^, our calculated GB adsorption energy shows near-zero values in all the embrittling couples; this outcome provides an energy criterion that determines the occurrence of embrittlement. On the other hand, surface adsorption must promote bond-breaking at a microcrack tip because surface energy reduction is well known to enhance temper embrittlement by solute segregation^12–16^ (Supplementary Note). However, our calculated surface adsorption energy of a liquid metal atom on solid metal does not correlate with the specificity of LME, as shown in Fig. 1b–d. So, what is the atomistic picture that explains these calculated results consistently?

**Table 1:** A specificity data of LME.

**Test temperature (℃)**													
	30	50	125	180	210	250	260	300	325	350	380	450	475
**Melting point temperature, *T***_**m**_** (℃)**													
	− 39	30	98	157	181	221	232	271	304	321	328	420	450
**Liquid metal**													
Solid metal	Hg*	Ga	Na	In	Li	Se	Sn	Bi	Tl	Cd	Pb	Zn	Te
Steel (Fe)	N	N	N	**E**	**E**	N	N	N	N	**E**	N	**E**	**E**
Al alloy	**E**	**E**	**E**	**E**	N	N	**E**	N	N	N	N	**E**	-
Mg alloy	N	N	**E**	N	N	N	N	N	N	N	N	**E**	-
Ti alloy	**E**	N	N	N	N	N	N	N	N	**E**	N	N	N

This table is taken from Rostoker et al.^10^ and modified. See text.

Hg* 3% Zn amalgam.

**E**: embrittlement with no apparent prior plastic deformation (probably GB cracking).

N: non-embrittlement.

Steel (Fe): The steel was an alloy constructional type quenched and tempered to Rc 45 hardness.

Al, Mg, and Ti alloys: The test was done using at least two high-strength commercial alloys for each metal.

**Figure 2:**
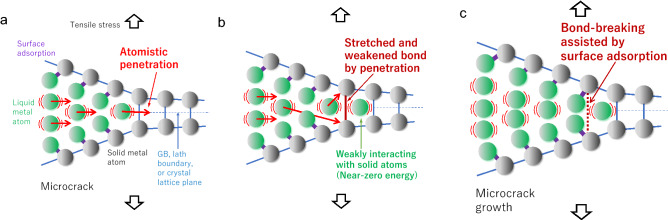
A proposed atomistic process of bond-breaking at a microcrack tip. (**a**) Atomistic penetration of liquid metal atom into GB, lath boundary, or lattice plane of solid metal at a microcrack tip under tensile stress. (**b**) The atomistic penetration of liquid metal with weak interaction (near-zero energy) stretches and weakens the solid metal bonding. (**c**) The subsequent surface adsorption of liquid metal atoms achieves the bond-breaking of solid metals. The process of (**a**–**c**) repeats, and the microcrack grows when the weak interaction criterion is satisfied.

**Figure 3:**
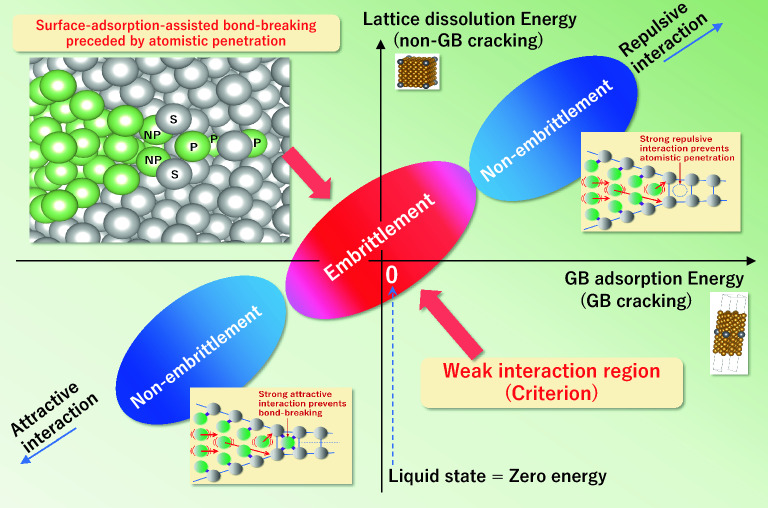
Schematic illustration of the criterion for the specificity of LME. P: a penetrated liquid metal atom into solid, NP: a not-yet-penetrated liquid metal atom that adsorbed on a solid metal atom, S: a solid metal atom with broken solid–solid interatomic bond by the adsorption of liquid metal atom. The solid–liquid couples that satisfy the weak interaction (near-zero energy) criterion show embrittlement. The atomistic embrittling (bond-breaking) process is schematically shown in Fig. 2 (Supplementary Fig. S9b), where the weak interaction criterion is satisfied. Contrarily, the atomistic penetration or the subsequent bond-breaking is suppressed in the cases of non-embrittlement (Supplementary Fig. S9ac).

We have further proposed an essential atomistic process necessary for bond-breaking at a microcrack tip, which controls the specificity of LME, as shown in Fig. 2. We consider that when embrittlement occurs without experimentally observing liquid metal atoms’ existence along the cracking path before fracture, it must be necessary for the liquid metal atom to penetrate at least one or several atomic distances and then be surrounded by weakly interacting solid metal atoms at a microcrack tip. The penetrated liquid metal atom probably stretches highly and weakens chemically solid–solid metal bonds across the GB, lath boundary in martensitic steel, and crystal lattice plane. Subsequently, not-yet-penetrated liquid metal atoms break the stretched and weakened interatomic solid metal bonds by adsorbing on these solid atoms. As a result, solid metal bond-breaking occurs irreversibly due to surface termination by liquid metal atoms, and therefore the microcrack grows. When the GB adsorption energy shows far-from-zero values, an intense repulsive or attractive interaction prevents the atomistic penetration or the subsequent bond-breaking, respectively, as shown in Fig. 3. Again to say, such an atomistic process may be impossible to be detected by modern experimental technology. Computational simulations will be necessary to make the process visible.For an experiment showing the specificity of LME, we paid particular attention to the result by Rostoker et al.^10^, as shown in Table 1. Although the experimental details are missing, this experiment conducted a bending test for more than 40 combinations (couples) under similar test conditions (except temperature). In this respect, this experiment is an incomparable one. The solid metals used are high-strength steel (Fe) and high-strength alloys of Al, Mg, and Ti, while liquid metals used are Hg, Ga, Na, In, Li, Se, Sn, Bi, Tl, Cd, Pb, Zn, and Te. According to the test results, solid–liquid couples were classified as either “Embrittlement” when a brittle fracture occurs with no apparent prior plastic deformation, probably GB cracking, or “Non-embrittlement” when such a severe brittle fracture does not occur. It is important to note that some non-embrittlement elements in Table 1 can cause embrittlement under other tests. For example, while Pb and Bi are non-embrittlement elements in Table 1, these liquid metals (Pb and Bi) cause embrittlement on T91 martensitic steel, where cracking occurs mainly along its lath boundaries with significant plastic deformation^17^.

To elucidate the mystery of the specificity of LME, we considered that the GB and surface adsorption energy of a liquid metal atom must be essential for the following reasons. In many cases, severe embrittlement occurs along GBs with almost no prior plastic deformation. Additionally, the (fracture) surface adsorption of a liquid metal atom must be necessary because the energy lowering effect by the adsorption can promote bond-breaking at a microcrack tip of solid metal. Based on these ideas, we calculated the GB and surface adsorption energies of a liquid metal atom in solid metals for many couples (Methods and Supplementary Fig. S2). The energies’ standard (zero value) for the liquid metal atom is the energy of its crystalline state at absolute zero temperature. It is a good approximation for the liquid state because the energy difference between the liquid and its solid states, i.e., the latent heat (enthalpy) of fusion (Supplementary Table S2)^18^, is insignificant compared with the size of the GB and surface adsorption energy.

As another factor, the interstitial lattice dissolution energy of the liquid metal atom in solid metal might be significant for the embrittlement along the low-energy lath boundary and crystal lattice plane. This type of embrittlement occurs with a large amount of prior plastic deformation, which is necessary to cause microcrack nucleation in non-GB cracking. Unfortunately, in our experiences, the interstitial lattice dissolution energy becomes too large in positive to obtain the numerically converged value. Although the tensile stress concentration at the microcrack tip is critical, it is not easy to include that in the calculations. For this reason, we calculated the substitutional lattice dissolution energy instead, which probably correlates to the interstitial one and lath boundary adsorption energy of a liquid metal atom in solid metal.

As stated in the fourth paragraph, we found an excellent correlation between the specificity of LME and our calculated GB adsorption energies of liquid metal elements in iron (Fe) and aluminum (Al), as shown in Fig. 1a–c. All the embrittling elements have near-zero GB adsorption energy, while non-embrittling elements have far-from-zero GB adsorption energy, except Al-Cd. For iron (steel), indium causes more GB embrittlement than lead and bismuth in Martin’s experiment^17^; indium consistently appears at near-zero energy more closely than lead and bismuth (Fig. 1a). It is also reasonable for sodium to have far-from-zero GB adsorption energy (Fig. 1a) because sodium rarely causes the embrittlement of steel^19^. The calculated GB adsorption energies of zinc in iron (Fig. 1a) and gallium in aluminum (Fig. 1c) are slightly negative values; these imply the zinc and gallium atoms feel weak attractive interaction from iron and aluminum GB, respectively, and can easily penetrate the GBs. Therefore, zinc diffusion in iron GB and liquid gallium penetration in aluminum GB would realize quickly^2–4^. In addition, we can see a similar trend as in iron and aluminum for both magnesium and titanium cases (Supplementary Fig. S3 and S4). However, there are only two embrittling couples for each, magnesium or titanium. Nickel also shows asimilar trend using other experimental data (Supplementary Note and Fig. S6). Therefore, the trend of near-zero GB adsorption energy for the embrittling couples must be a necessary condition and criterion for the occurrence of LME.

Furthermore, we found an exciting trend; a continuous view from the calculated energies, as shown in Fig. 1a–c (Supplementary Fig. S3, S4, and S6), indicates that the embrittlement seems to occur in a transition region from where the intermetallic compounds exist to where they do not exist (Supplementary Note). The greater the GB adsorption and lattice dissolution energies are negative, the more the solid–liquid couple forms intermetallic compounds. Rostoker et al. pointed out that the embrittling couples tend not to form intermetallic compounds^10^, while our criterion further narrowed down the allowed range of embrittling couples from the viewpoint of interaction energy.

Lowering the cohesive energy of the GB plane caused by surface adsorption by liquid metal can promote interatomic bond-breaking in solid metal as in temper embrittlement^12–16^ (Supplementary Note). Figure 1b–d shows the calculated surface adsorption energies of liquid metal atoms on iron’s (111) and aluminum’s (012) surfaces. The surface adsorption energies were substantial negative values in all the couples, indicating that all liquid metal elements decrease the surface energy by adsorption and thus assist bond-breaking. However, there seems to be no correlation between the size of surface adsorption energy and the specificity of LME. It indicates that the condition of near-zero GB adsorption energy is more significant for LME than the surface adsorption strength. We expect the trend in Fig. 1b–d not to change if we could calculate solid–liquid interface energy because the cohesion of low melting temperature metal may not be significant. Finally, the above energetic considerations have led us to the following atomistic picture.

We explain the atomistic process of bond-breaking of solid metal induced by liquid metal atoms at a microcrack tip (Fig. 2) in more detail than already stated in the fifth paragraph. When embrittlement occurs, the GB adsorption energy of a liquid metal atom is near zero; the liquid metal atom weakly interacts with the surrounded solid atoms. Thus, the liquid metal atom can penetrate inside the microcrack tip at least one or several atomic distances through the stretched solid metal’s interatomic bonds under tensile stress concentration with the help of thermal activation. This atomistic penetration of the liquid metal must have some weakening effects on solid–solid bonds: a highly stretching effect and a chemical effect due to the change of atoms’ arrangement. Then, not-yet-penetrated liquid metal atoms adsorb the solid metal atoms consisting of the weakened interatomic bonds and then cause the bond-breaking at the microcrack tip. Although the energy reduction by surface adsorption can promote bond-breaking, the strength of surface adsorption does not correlate with the specificity of LME, as shown in Fig. 1b–d. Therefore, we conclude that the atomistic penetration of a liquid metal atom into solid metal’s GB plane is necessary before surface-adsorption-assisted bond-breaking occurs. Even when we cannot find liquid metal atoms experimentally along the cracking path before fracture, we consider that the atomistic penetration of liquid metal atoms precedes the bond-breaking of solid metal. Further, we consider that a similar atomistic penetration and the subsequent bond-breaking can occur at low-energy boundaries like lath in martensitic steel and crystal lattice planes, after significant prior plastic deformation and subsequent microcrack nucleation having huge stress concentration at its tip in the case of non-GB cracking.

On the other hand, if the atomistic penetration and subsequent diffusion of liquid metal atoms into GBs are experimentally observed, as in the case of galvanized (Zn) embrittlement of high-strength steel, the embrittlement is triggered by grain boundary decohesion at a first stage. We can recognize a significant decohesion effect of Zn on Fe GB in our calculated results; the surface adsorption energy of Zn (–0.8 eV/atom) is much lower than the GB adsorption energy of Zn (–0.3 eV/atom), which data can be found in Fig. 1a,b. This energy difference assists bond-breaking and surface formation in solid metal’s GB. Recent experimental and theoretical studies confirm this decohesion mechanism^4,20^ in more detail. We emphasize here that our proposed criterion of LME includes this type of embrittlement. When liquid metal atoms can penetrate and diffuse into solid metals’ GB macroscopically, the decohering effect on GB will emerge for almost all liquid metals because they have a significant surface adsorption effect stronger than GB adsorption in solid metals. (Supplementary Fig. S10).

When the weak interaction (near-zero energy) criterion of atomistic penetration is not satisfied, we consider the bond-breaking does not occur for the following reasons, as shown in Fig. 3 (Supplementary Fig. S9ac). When the GB adsorption energy of liquid metal is enormously positive, the liquid metal atom cannot penetrate the GB plane even at a microcrack tip because of the intense repulsive interaction between liquid and solid atoms. When the GB adsorption energy is significantly negative, the attractive interaction (bonding) is so intense between solid and liquid atoms that it prevents solid–solid bond-breaking near the liquid atom, even if the atomistic penetration occurs. Therefore, the bond-breaking (embrittlement) does not occur when the GB adsorption energy appears in the far-from-zero energy region, where the weak interaction criterion is not satisfied. The boundary between embrittlement and non-embrittlement regions depends on various material and test parameters such as hardness, grain size, temperature, strain rate, etc.

We should note our idea for the role of dislocations on LME. It comes from the analogy of the study of GB decohesion by one of the authors (MY)^16^. We consider that the easier the bond-breaking occurs at a microcrack tip, the lesser the dislocations emit from there^14,15^, reducing fracture toughness and fracture stress in LME. The present study assumes that surface and GB adsorption itself does not affect the dislocation emission because the influence of liquid metal on dislocation is not evident theoretically and experimentally. Further investigation is necessary on this point.Finally, we note that our study is not applicable for a different type of LME; it is primarily a dissolution or corrosion reaction, the crack growth takes hundreds or thousands of hours. Recently, Norkett et al.^11^ have classified LME into three categories: (a) solid metal seems to be brittle simply by touching liquid metal (e.g., Fe-Hg, Fe-Pb, Fe-Bi, and Zn-Hg)^6–8^, (b) liquid metal atoms diffuse as an atom or penetrate as a liquid along the GB plane of solid metal over a macroscopic distance (e.g., Fe-Zn, Al-Ga, and Ni-Bi)^2–5^, and (c) GBs are selectively eroded by corrosion reactions (e.g., a preferential dissolution of Cr and Ni from stainless steel in molten sodium)^21^. Our proposed weak interaction criterion based on experimentally unseen atomistic penetration adapts well to type (a) embrittlement. Furthermore, this criterion is also applicable to type (b) embrittlement; the penetration of liquid atoms surrounded by weakly interacting solid atoms is necessary before it develops to a macroscopic diffusion of atoms, interfacial phase, or liquid penetration, and then fracture occurs. On the other hand, the criterion does not adapt to type (c) embrittlement due to corrosion and dissolution reactions, in which solid metal atoms dissolve into liquid metal.

## Methods

### First-principles calculations

We conducted density functional theory (DFT) calculations by using Vienna Ab initio Simulation Packages (VASP)^23,24^ using the Projector-Augmented Wave method with Perdew-Burke-Ernzerhof exchange–correlation (PAW-PBE) potentials^25,26^. The cutoff parameter of the plane-wave basis set was 360 eV. (For the Cu system, we use 385 eV).

We calculated the substitutional lattice dissolution (solid solution) energy of liquid metal elements in the solid metal lattice using supercells. Supplementary Table S1 summarizes solid metal crystal structures, magnetism, supercell size, and k-point sampling. The total energy of superlattice having *n* solid metal atoms is ${E}_{\mathrm{tot}}^{\mathrm{sc}}(n)$, and that having $n-1$ solid metal (M) atoms and one X atom ${E}_{\mathrm{tot}}^{\mathrm{sc}}(n-1, \mathrm{X})$. The total energy per one liquid metal atom in the most stable elemental substance is ${E}_{\mathrm{atom}}(\mathrm{X})$. Supplementary Table S2 shows the crystal structure of each liquid metal in low temperatures. These cells were fully relaxed. The lattice dissolution energy of element X in a solid metal is defined by.

1 $${E}_{\mathrm{sol}}\left(\mathrm{X}\right)={E}_{\mathrm{tot}}^{\mathrm{sc}}\left(n-1,\mathrm{ X}\right)-{\frac{n-1}{n}E}_{\mathrm{tot}}^{\mathrm{sc}}\left(n\right)-{E}_{\mathrm{atom}}(\mathrm{X})$$

We calculated the grain boundary and surface adsorption energies of more than ten liquid metal elements in five solid metals. Supplementary Table 1 shows the details of cell modeling for solid metals. The unit cells, including grain or twin boundary and its two fracture surfaces, are shown in Supplementary Fig. S1. Supplementary Table S3 summarizes the calculated grain boundary and surface energies. The grain or twin boundary energy is about half of the surface energy for each solid metal except for titanium. Therefore, it indicates that these boundaries are high-energy ones. On the other hand, the boundary energy is relatively low for titanium. It may be why the calculated adsorption energies of grain boundary and surface correlate very well with each other in the titanium case, as shown in Supplementary Fig. S4.

The total energy of these cells is denoted by ${E}_{\mathrm{tot}}^{\mathrm{GB}}(n)$. We relaxed the atomic positions with fixed lattice parameters. The grain-boundary and surface adsorption energy ${E}_{\mathrm{ad}}(\mathrm{X})$ is defined by.

2 $${E}_{\mathrm{ad}}\left(\mathrm{X}\right)={E}_{\mathrm{tot}}^{\mathrm{GB}}\left(n,\mathrm{ X}\right)-{E}_{\mathrm{tot}}^{\mathrm{GB}}\left(n\right)-{E}_{\mathrm{atom}}\left(\mathrm{X}\right),$$

or

3 $${E}_{\mathrm{ad}}\left(\mathrm{X}\right)={E}_{\mathrm{tot}}^{\mathrm{GB}}\left(n-1,\mathrm{ X}\right)-{E}_{\mathrm{tot}}^{\mathrm{GB}}\left(n\right)+{E}_{\mathrm{atom}}\left(\mathrm{M}\right)-{E}_{\mathrm{atom}}\left(\mathrm{X}\right).$$

The former and latter are in the cases of interstitial and substitutional X atoms, respectively. The energy per atom for solid metal M, ${E}_{\mathrm{atom}}(\mathrm{M})$, is calculated by using a cell similar to the grain-boundary modeling cell to reduce numerical inaccuracy. The most stable states are selected for GB and surface adsorption energies at GB and surface, respectively.

When dealing with segregation in the atomistic model of the grain boundary, there are two equations to calculate the segregation energy, i.e., interstitial or substitutional type for the Eqs. (2) or (3), respectively. However, in the case of metal grain boundaries, the grain boundary energy often does not change significantly in response to the insertion or removal of the atoms that construct the grain boundary. The distinction between substitutional and interstitial types is not meaningful for the segregation of solute elements in disordered structures of metal GB. For this reason, we choose the lower GB adsorption energy from the above two equations.

Supplementary Fig. S2 shows an example of the calculated grain-boundary and surface adsorption energies of a Pb atom in the solid metal of iron. We can see that the lattice dissolution energy calculated by using the BCC 5 × 5 × 5 supercell is close to the corresponding energies calculated by using the grain boundary modeling cell. Thus, it indicates that the cell size we used is sufficient. As explained below, we take the fcc solid-state energy of Pb as an approximation of its liquid state energy.

### Energies of liquid state

All of the liquid metals examined here are low-melting-point metals. Thus, the latent heat, the difference in energy between solid and liquid, is on the order of at most 0.02–0.18 eV/atom^18^, as shown in Supplementary Table S2, which is not significant compared with the scale of lattice dissolution and surface adsorption energies. For example, the Pb energy difference between solid and liquid states is only 0.05 eV/atom at melting temperature. This energy difference is too small to influence the interpretation of the calculated results (Fig. 2). For this reason, we consider that there is no problem adopting the solid-state energy of the liquid metal element as an approximation of the liquid state energy.

## Supplementary Information

Supplementary Information 1.

Supplementary Information 2.

## Data Availability

All data are available in the main text or supplementary information.
