# Supplementary material for: Atomistic weak interaction criterion for the specificity of liquid metal embrittlement
Source: Sci Rep. 2022 Jul 4;12:10886. doi: 10.1038/s41598-022-10593-2 (PMC9253112; doi:10.1038/s41598-022-10593-2)
Supplement: Supplementary file 1 — Supplementary Information 1. [file 41598_2022_10593_MOESM1_ESM.pptx]

## Slide 1
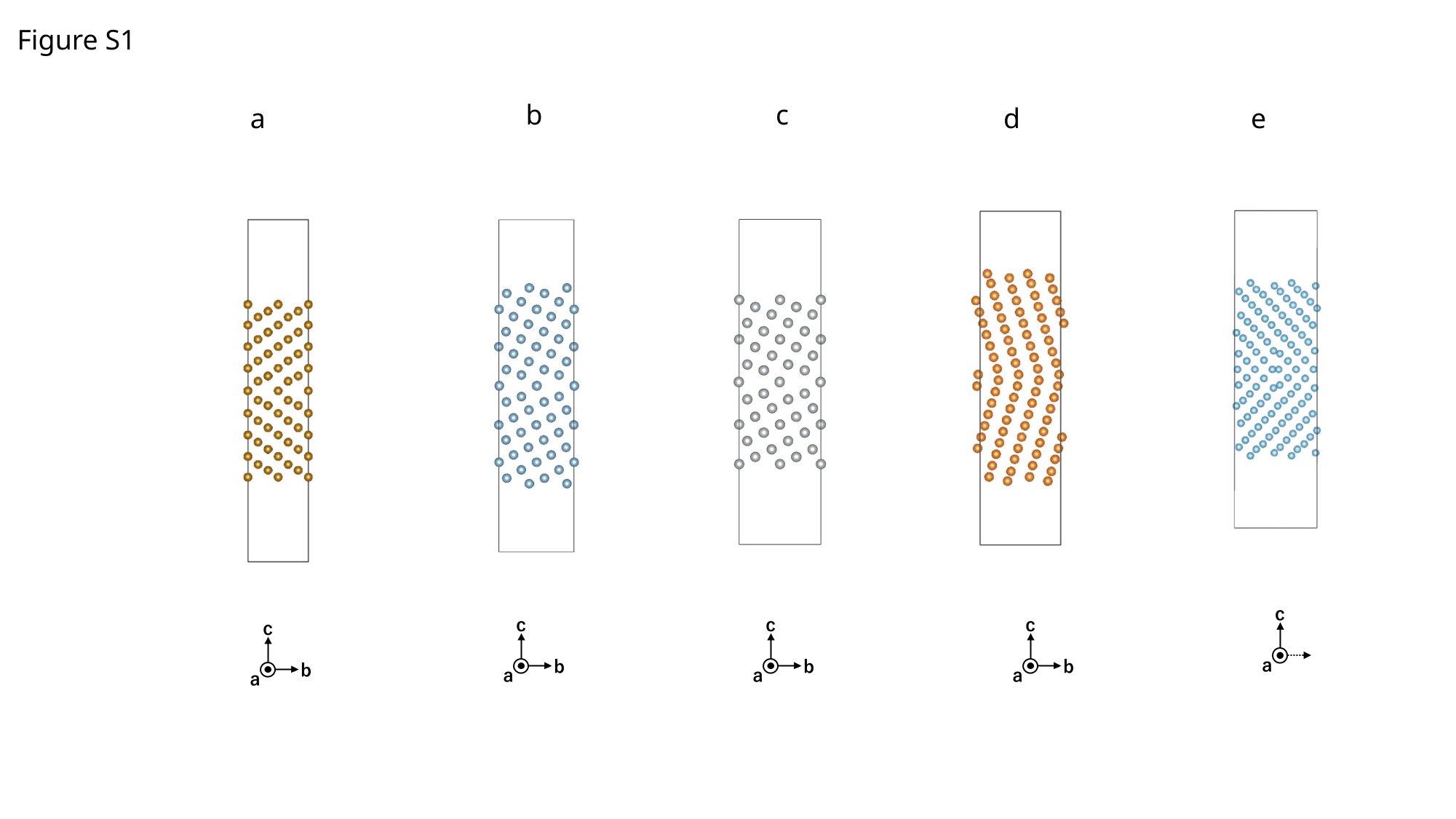

Figure S1
b
c
a
d
e

## Slide 2
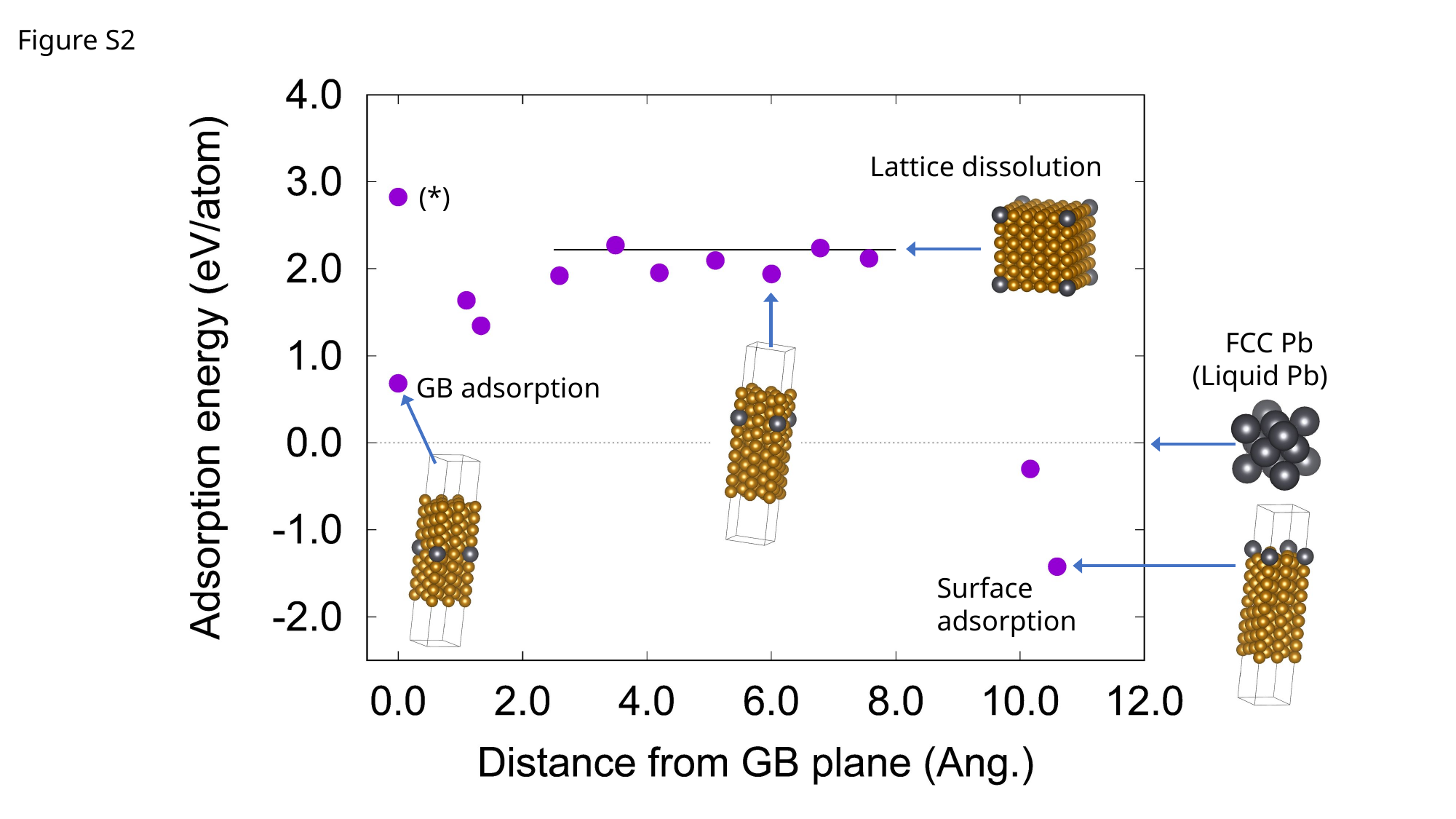

Figure S2
Lattice dissolution
(*)
FCC Pb
(Liquid Pb)
GB adsorption
Surface
adsorption

## Slide 3
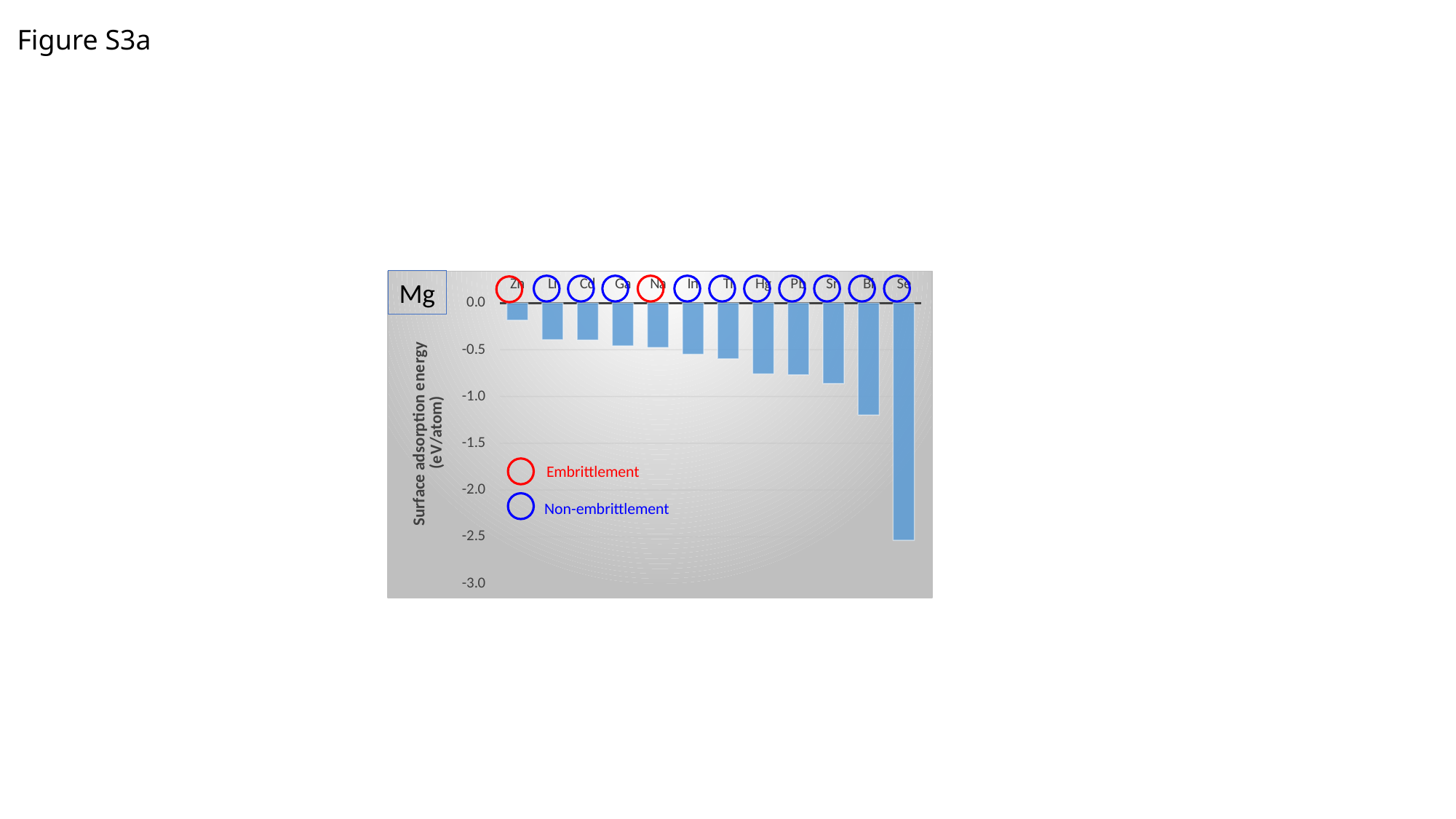

Figure S3a
### Chart
| Category | |
|---|---|
| Zn | -0.185 |
| Li | -0.3919 |
| Cd | -0.3971 |
| Ga | -0.458585 |
| Na | -0.47635 |
| In | -0.5487 |
| Tl | -0.5960000000000001 |
| Hg | -0.7586499999999999 |
| Pb | -0.7681 |
| Sn | -0.8615999999999999 |
| Bi | -1.198 |
| Se | -2.5382 |
Embrittlement
Non-embrittlement
Mg

## Slide 4
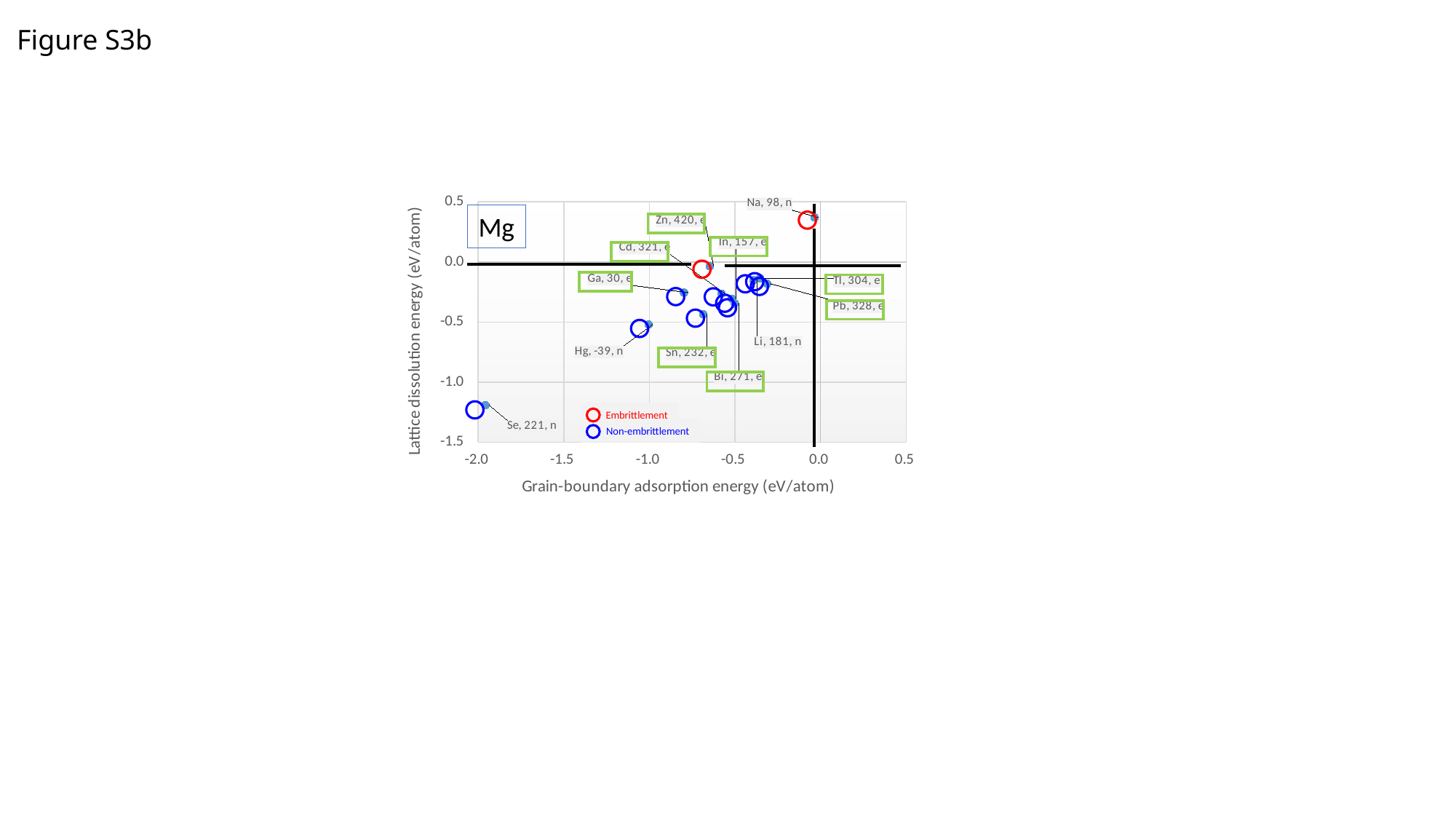

Figure S3b
### Chart
| Category | |
|---|---|Mg
 Embrittlement
 Non-embrittlement

## Slide 5
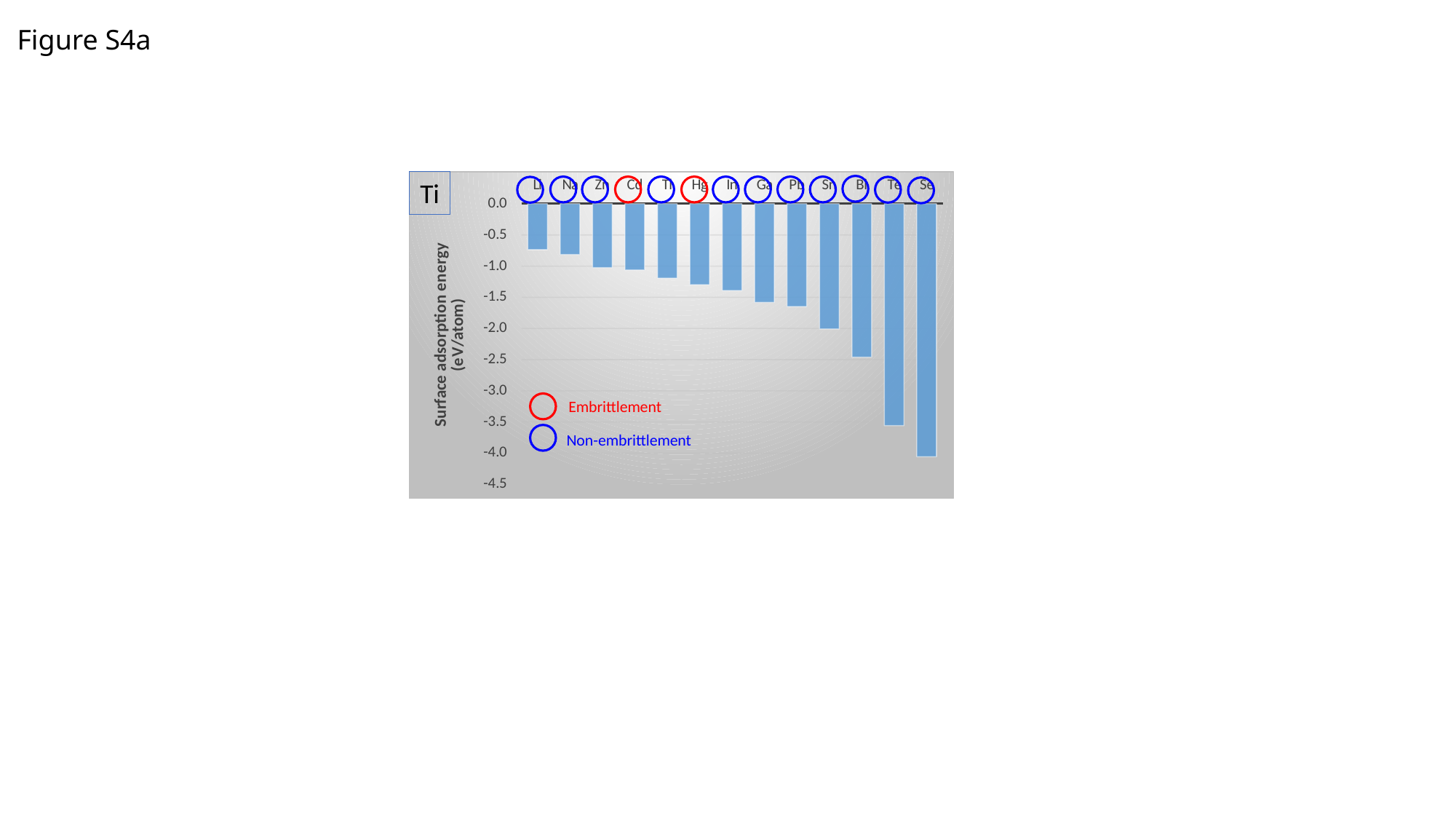

Figure S4a
### Chart
| Category | |
|---|---|
| Li | -0.736 |
| Na | -0.8155000000000001 |
| Zn | -1.0235 |
| Cd | -1.0627 |
| Tl | -1.1923000000000001 |
| Hg | -1.2993025999999999 |
| In | -1.3927 |
| Ga | -1.583 |
| Pb | -1.649 |
| Sn | -2.0089 |
| Bi | -2.4606 |
| Te | -3.558 |
| Se | -4.057 |
Embrittlement
Non-embrittlement
Ti

## Slide 6
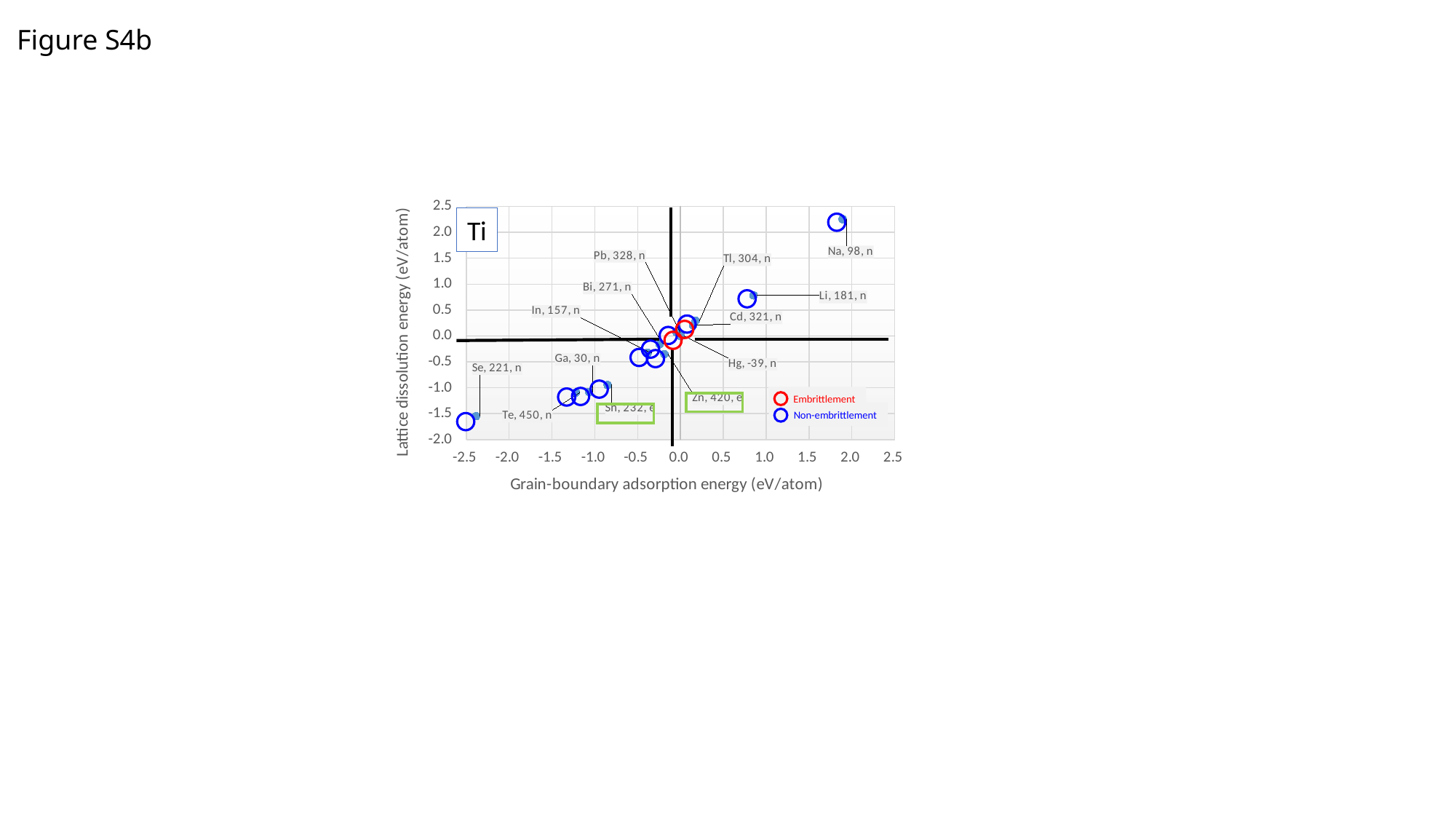

Figure S4b
### Chart
| Category | |
|---|---|Ti
 Embrittlement
 Non-embrittlement

## Slide 7
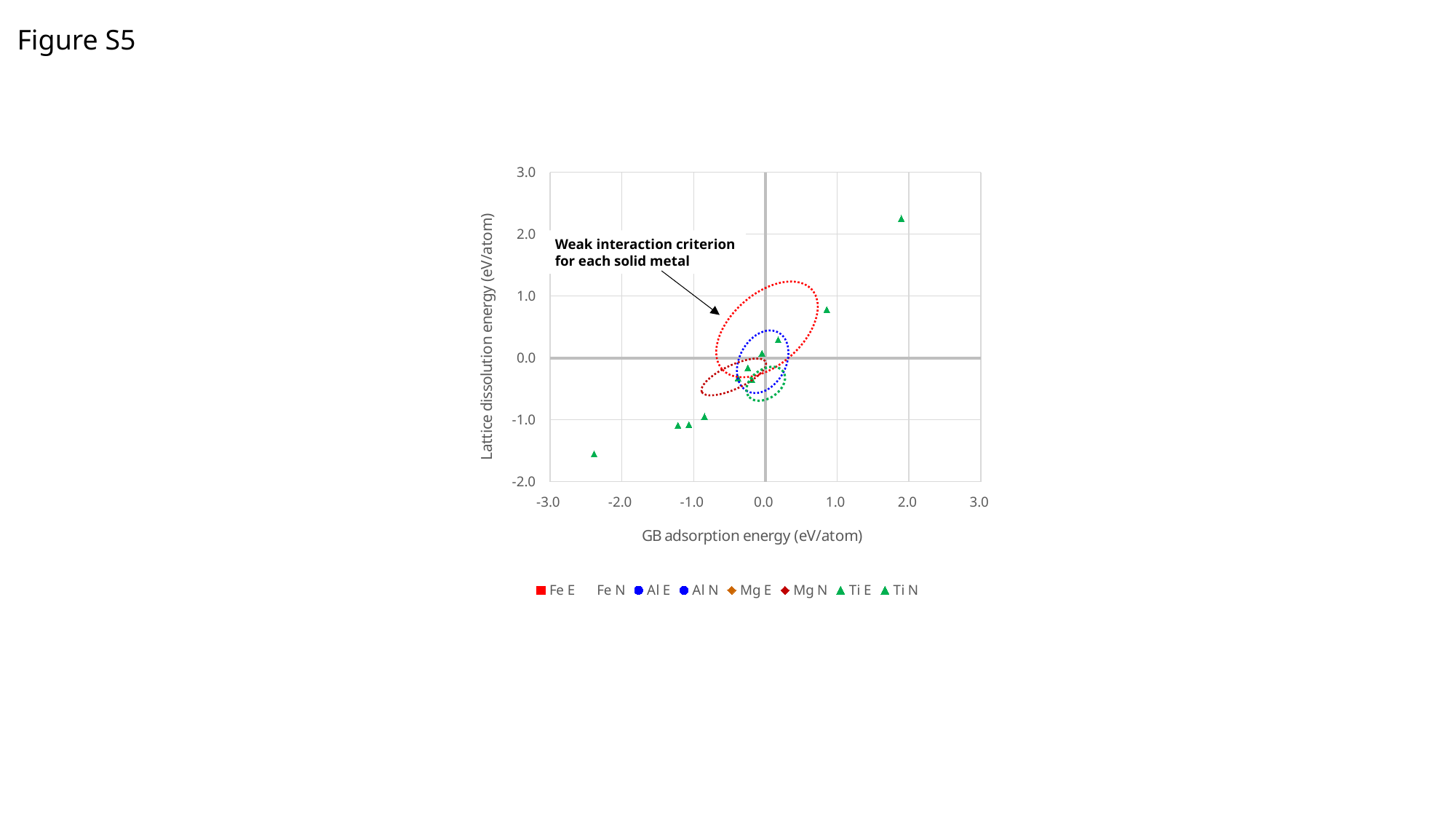

Figure S5
### Chart
| Category | | | | | | | | |
|---|---|---|---|---|---|---|---|---|Weak interaction criterion
for each solid metal

## Slide 8
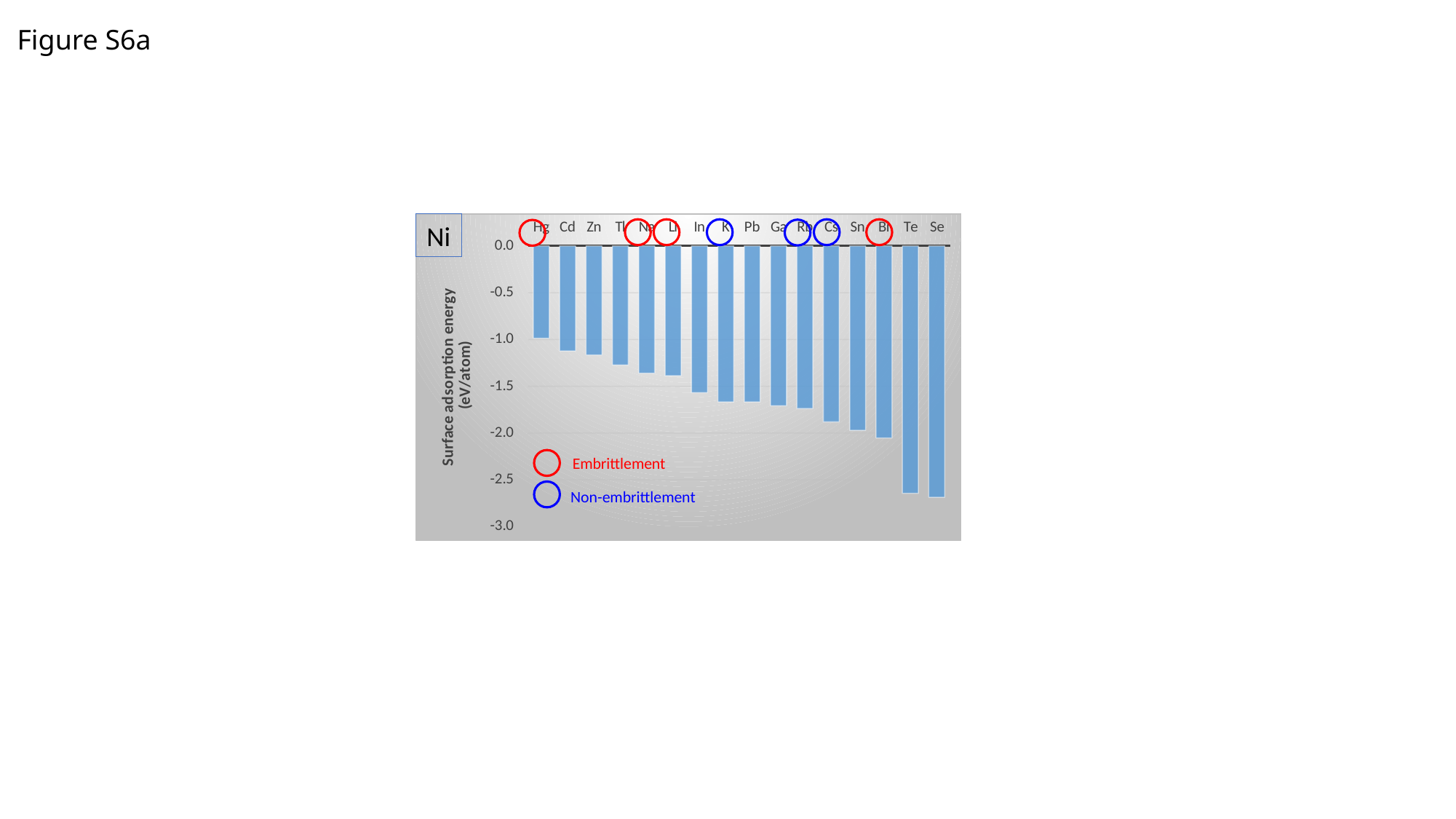

Figure S6a
### Chart
| Category | |
|---|---|
| Hg | -0.9865999999999997 |
| Cd | -1.122685 |
| Zn | -1.16563 |
| Tl | -1.2737999999999998 |
| Na | -1.3611 |
| Li | -1.387 |
| In | -1.56846 |
| K | -1.6678300000000004 |
| Pb | -1.6688999999999998 |
| Ga | -1.7087432000000002 |
| Rb | -1.7392000000000003 |
| Cs | -1.8812000000000006 |
| Sn | -1.9716 |
| Bi | -2.0534 |
| Te | -2.645771 |
| Se | -2.686851 |
Embrittlement
Non-embrittlement
Ni

## Slide 9
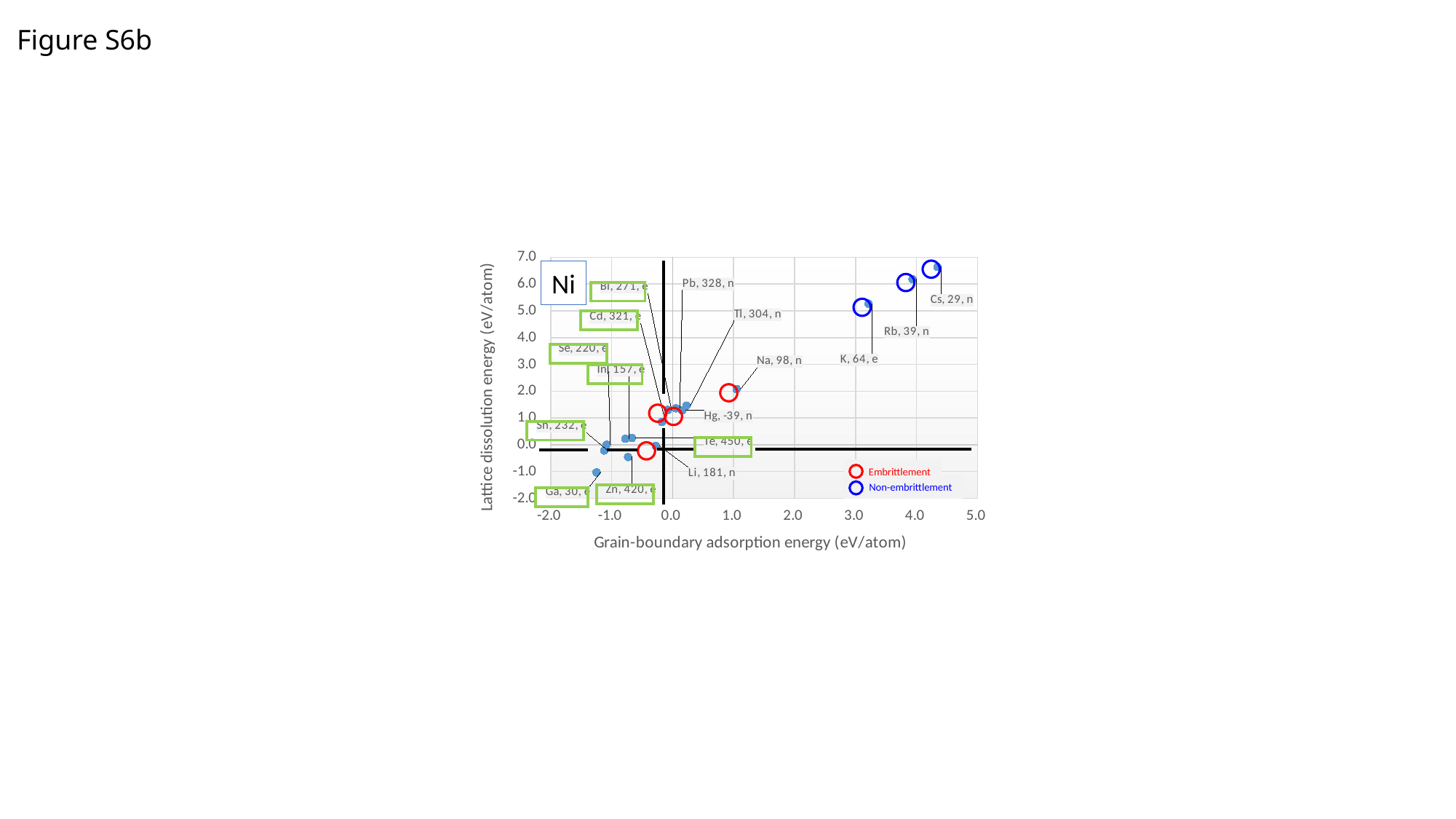

Figure S6b
### Chart
| Category | |
|---|---|
 Embrittlement
 Non-embrittlement
Ni

## Slide 10
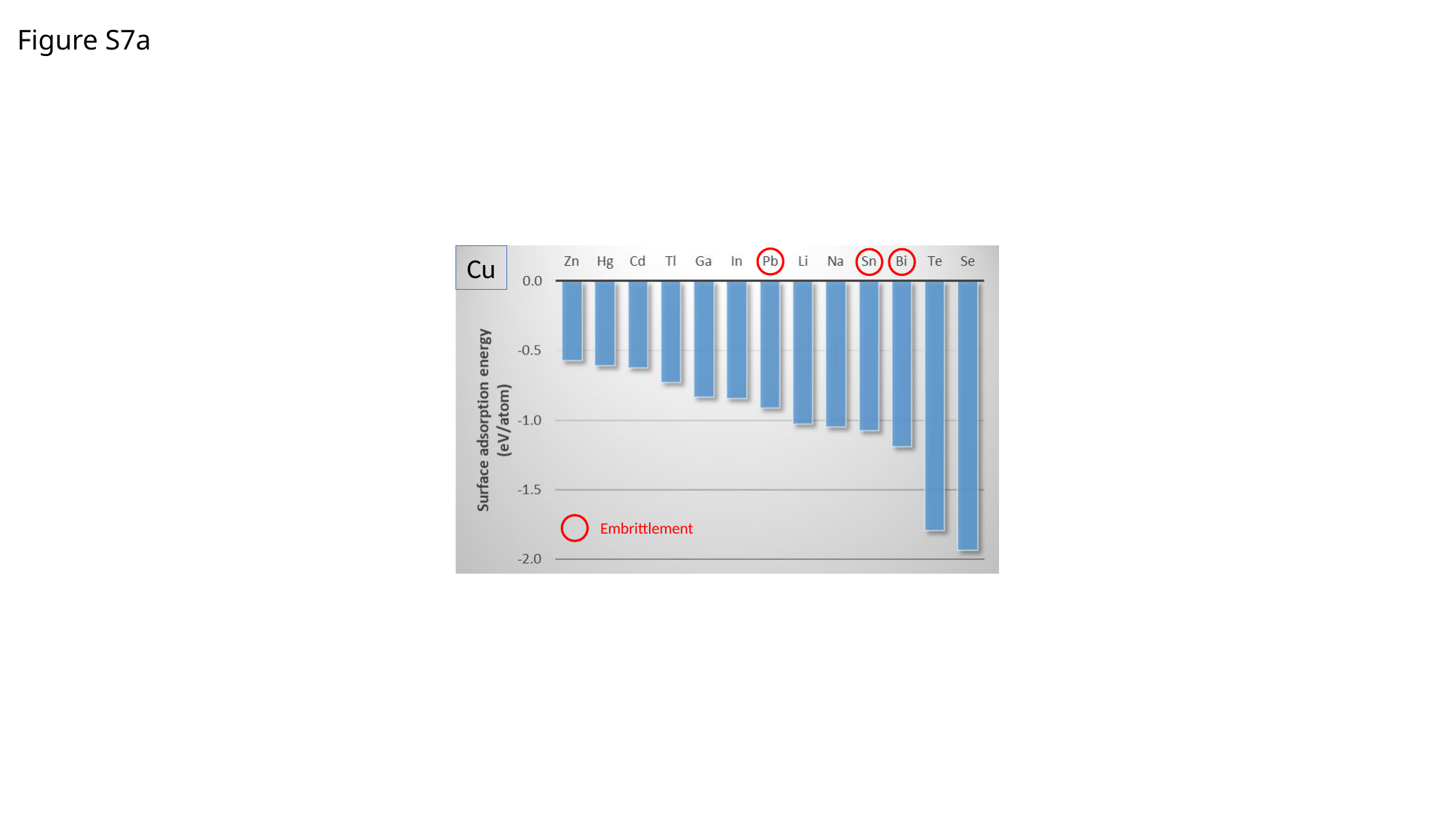

Figure S7a
Cu
Embrittlement

## Slide 11
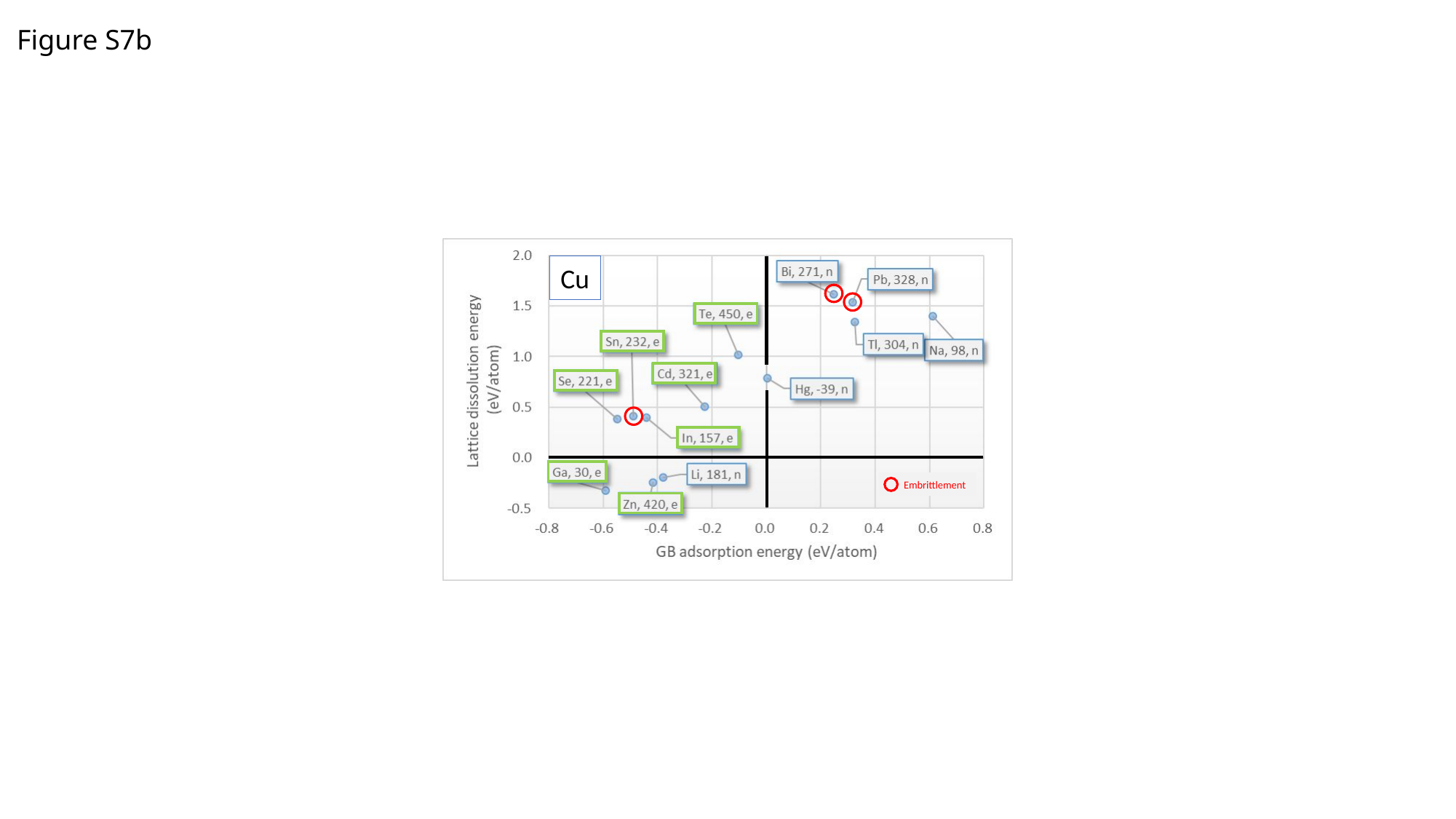

Figure S7b
Cu
 Embrittlement

## Slide 12
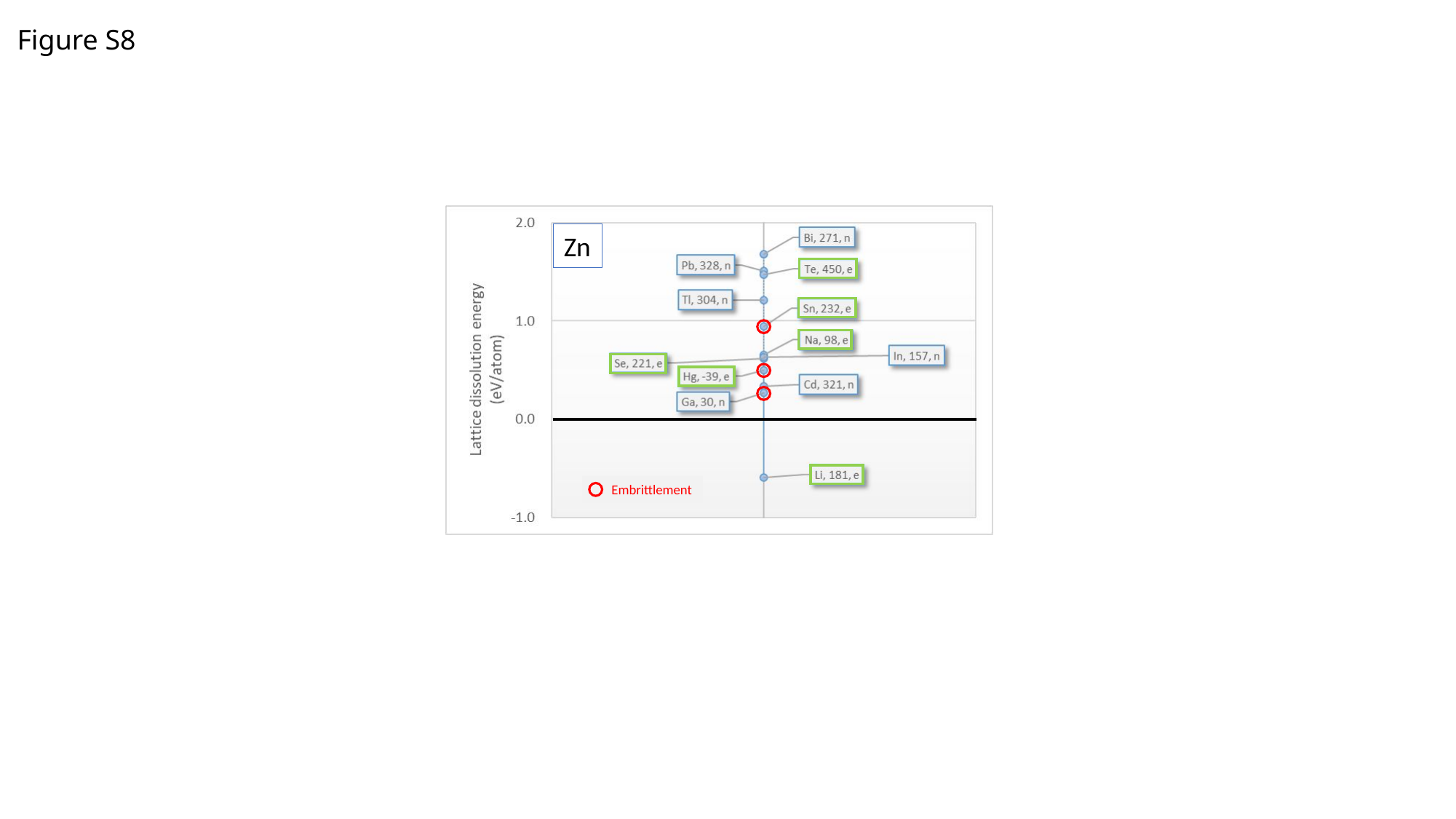

Figure S8
Zn
 Embrittlement

## Slide 13
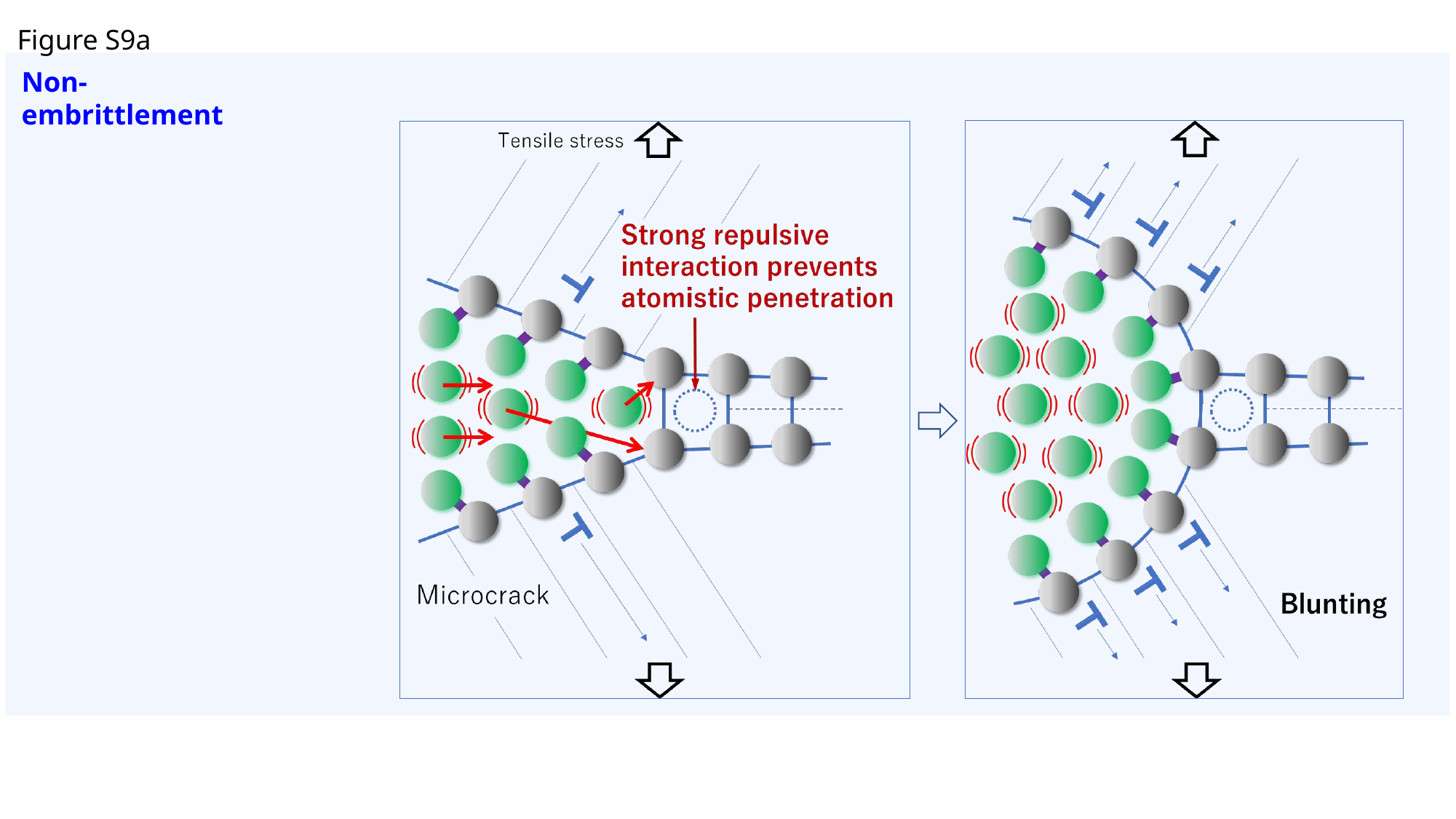

Figure S9a
Non-embrittlement

## Slide 14
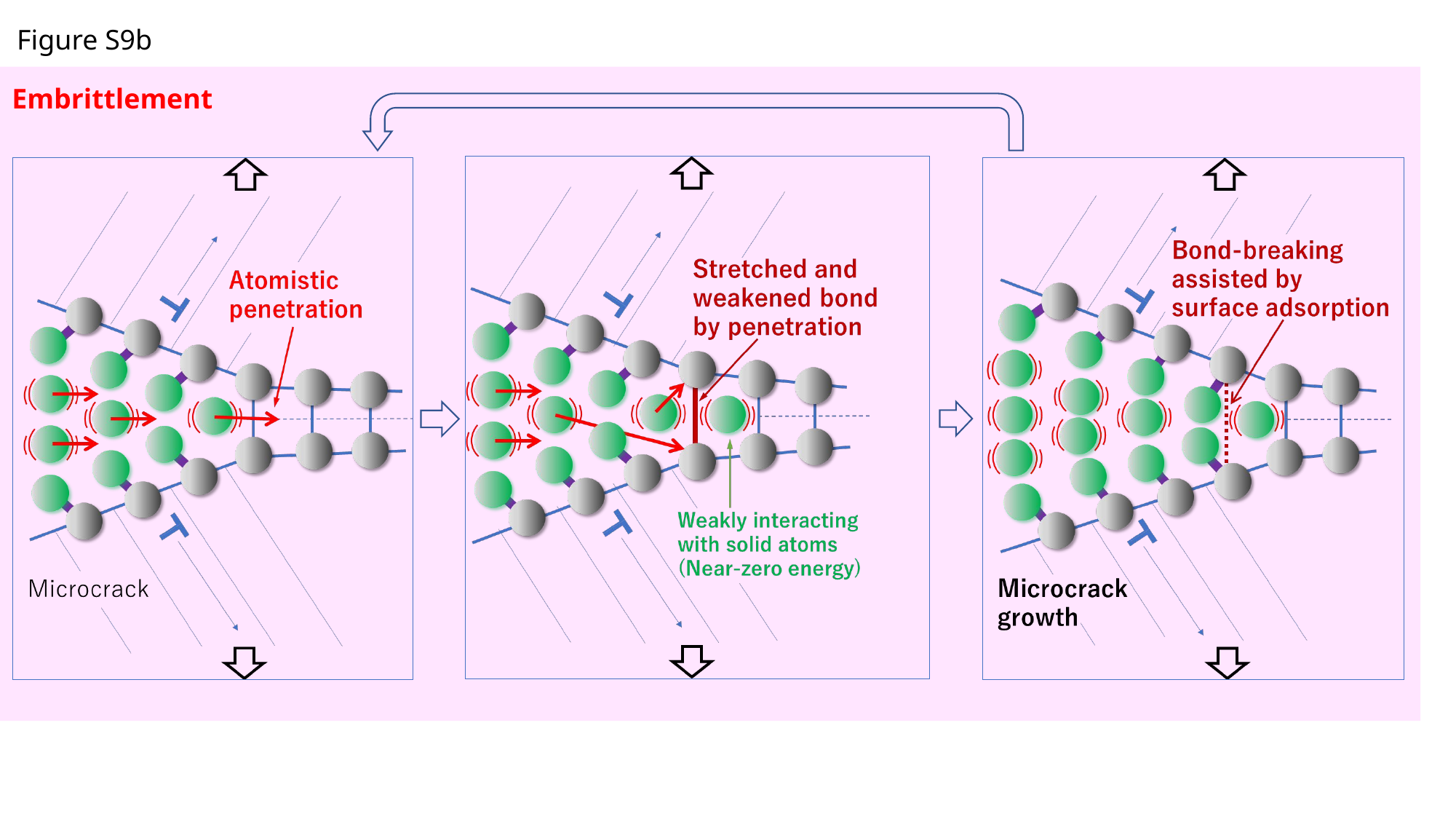

Figure S9b
Embrittlement

## Slide 15
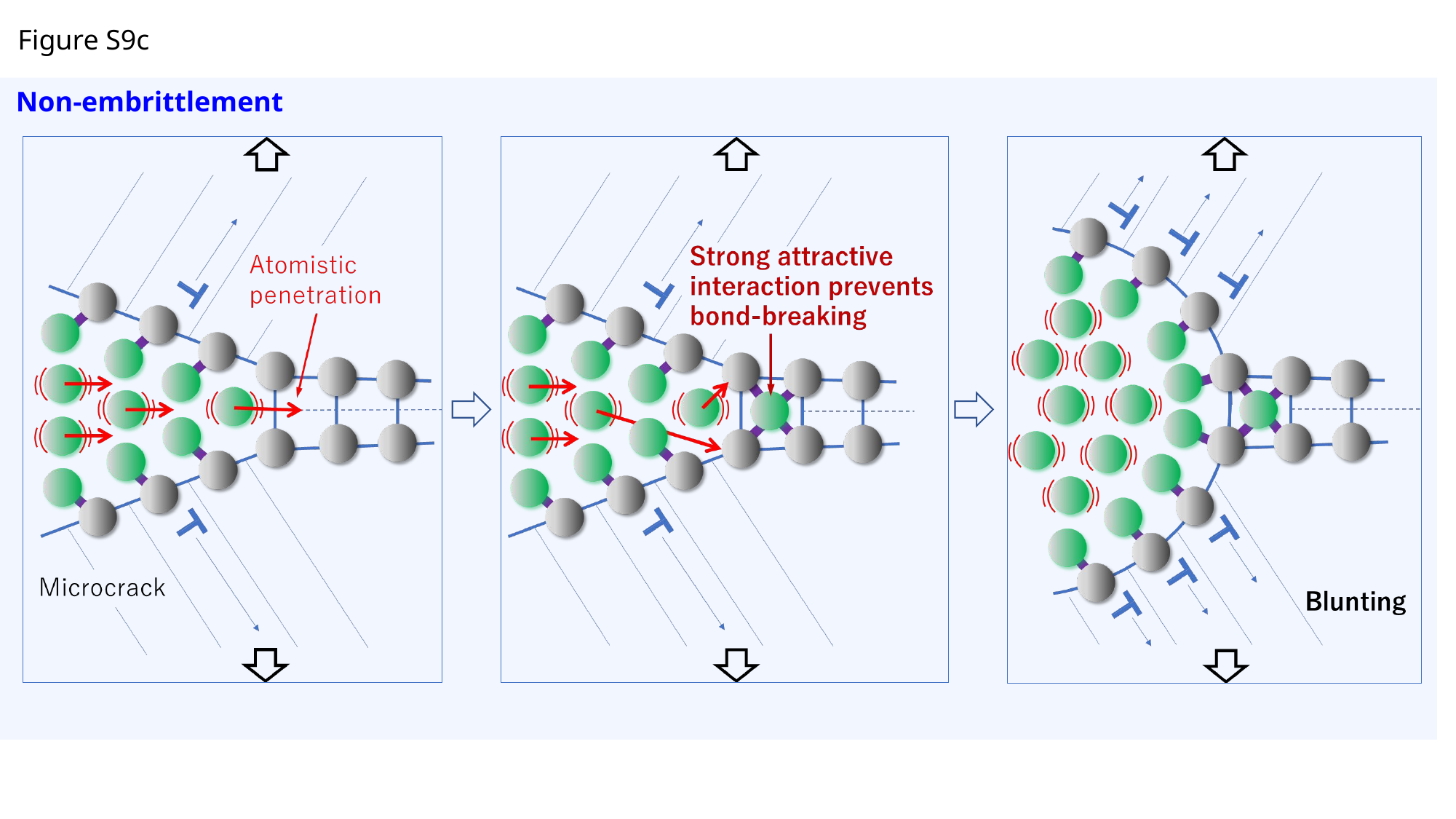

Figure S9c
Non-embrittlement

## Slide 16
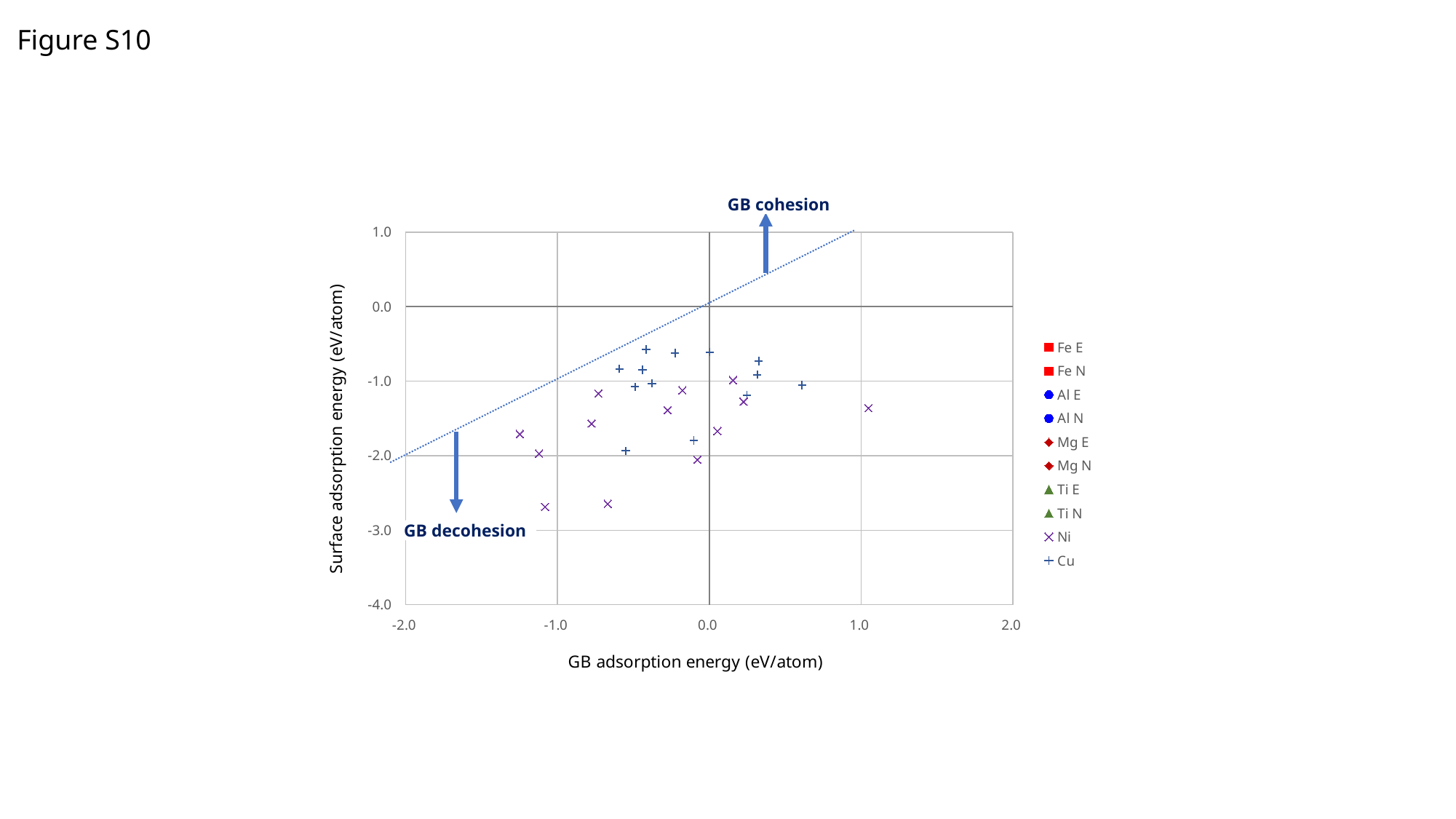

Figure S10
GB cohesion
### Chart
| Category | | | | | | | | | | |
|---|---|---|---|---|---|---|---|---|---|---|GB decohesion
