# Supplementary material for: Atomistic weak interaction criterion for the specificity of liquid metal embrittlement
Source: Sci Rep. 2022 Jul 4;12:10886. doi: 10.1038/s41598-022-10593-2 (PMC9253112; doi:10.1038/s41598-022-10593-2)
Supplement: Supplementary file 2 — Supplementary Information 2. [file 41598_2022_10593_MOESM2_ESM.docx]

Supplementary Information for

Atomistic Weak Interaction Criterion for the Specificity of Liquid Metal Embrittlement

Masatake Yamaguchi, Tomohito Tsuru, Mitsuhiro Itakura, Eiji Abe

Correspondence to: [yamaguchi.masatake@jaea.go.jp](mailto:yamaguchi.masatake@jaea.go.jpx)

**This PDF file includes:**

Supplementary Note

Reference 23 to 29

Table S1 to S3

Figure S1 to S10

Supplementary Note

Liquid metal embrittlement (LME) of high-strength steels (Fe)

Although the results of LME specificity vary widely depending on the materials and test conditions, the positions of the liquid metal elements that appeared in Fig. 1a are consistent with and convincing to many specific experimental results. Some examples are as follows.

Legris et al.’s experiment^27^ indicate that Pb-Bi eutectic, Sn, and Hg embrittles high-strength T91 steel, while Rostoker et al.’s experiment^2^ shown in Fig. 1a indicates that they belong to the non-embrittlement group. However, the amount of deformation before fracture is large in Legris et al.’s result while small in Rostoker et al.’s one. Therefore, it is reasonable that these elements appear near the boundary of the embrittlement group in Fig. 1a.

Martin et al.’s experiment^17^ indicate that indium, lead, and bismuth embrittle high-strength T91 steel. It is interesting to see that indium embrittles the steel most severely in their experiment. Although the fracture mode is almost transgranular, the intergranular fracture can be seen only in the case of indium. The indium position in Fig. 1A belongs to the embrittlement group while lead and bismuth to the non-embrittlement group. Therefore, the trend of embrittlement for lead, bismuth, and indium in Martin et al.’s experiment is well understood from our calculated results, as shown in Fig. 1a.

Lynch also shows that the D6ac steel shows various types of LME fracture by Hg^6^. Hg appears near the boundary between the embrittlement and non-embrittlement groups in Fig. 1a. The fracture morphology varies from ductile to brittle fracture, depending on heat treatment. We cannot mention the mechanism of embrittlement insisted by Lynch, the adsorption-induced dislocation emission (AIDE) mechanism, because it is not easy to investigate the dislocation emission from the solid metal surface and the effect of adsorption on it by using atomistic simulations.

Molten Zn embrittles high-strength steels^3^ for both ferritic and austenitic ones. Some observations show the diffusion of the Zn atom into steel grain boundaries ahead of the crack tip. Therefore, it is reasonable that the position of Zn in Fig. 1a is very close to zero value which is the center of the region.

Sodium (Na) has been well investigated and known to embrittle steels rarely^19^. The position of sodium appearing in Fig. 1a is reasonable because it implies that Na does not embrittle Fe compared with lead and bismuth.

LME of aluminum (Al)

We can see a good correlation, as shown in Fig. 1c. The only exception is cadmium. Rostoker et al.’s experiment^1^ indicated that cadmium was a non-embrittlement element. However, some papers describe Al-Cd as an embrittlement couple^9,28^. One experiment has shown that adding three at% cadmium to mercury enhances the embrittlement of pure aluminum^28^. It suggests that cadmium may cause more embrittlement to aluminum than mercury. In Fig. 1c, cadmium is closer to zero energy than mercury (Hg). It is a consistent result. In addition, the couples that tend to form intermetallic compounds are concentrated only in the lower-left part of Fig. 1c. As seen in Fig. 1a for iron, the continuous view in the trend of intermetallic compound formation correlates with the specificity of LME for aluminum.

As mentioned in the main text, the embrittlement by gallium in the polycrystalline aluminum alloy is the most remarkable phenomenon in LME. In polycrystalline aluminum, liquid gallium penetrates the aluminum grain boundary at high speed, about 0.26 μm/sec at room temperature^2^. Thus, liquid gallium penetrates with breaking the grain interface. However, on the other hand, the embrittlement occurs even in its single crystal at room temperature.

Gallium appears much closer to zero energy than other elements on the energy map of Fig. 1b. Therefore, in the case of single-crystal aluminum, gallium atoms can atomistically penetrate the microcrack tip in aluminum lattice under applied stress and not stabilize so firmly. Then, the surface adsorption effect observed in Fig. 1d can promote fracture surface formation.

On the other hand, in polycrystalline aluminum alloys, the energy of the aluminum-gallium solid-liquid interface is probably less than 1/2 of the energy at the aluminum grain boundary, and the penetration of gallium atoms achieves cracking of aluminum grain boundaries without the aid of stress. In other words, in the form of embrittlement in which liquid metal develops rapidly at grain boundaries, it can be considered that the interatomic bond breaking at the microcrack tip is achieved without the aid of external stress. Norkett et al. discussed the grain-boundary diffusion and wetting behavior related to LME^11^.

LME of magnesium (Mg) and titanium (Ti)

Fig. S3 and S4 show the results of magnesium and titanium alloys, respectively. The liquid metal elements that caused embrittlement in Rostoker et al.’s experimental results^10^ are near-zero energy, and there is no contradiction. Although some elements close to zero energy do not cause embrittlement, other factors may prevent embrittlement. Unfortunately, the details of the experiment are unknown.

In the case of Mg, almost all liquid metals are located in the negative region from zero energy, suggesting that the chemical bonding between liquid metals and magnesium atoms is strong in lattice and grain boundaries. On the other hand, in titanium, the liquid metal elements of cadmium and mercury, which cause LME, are located at almost zero energy.

The correlation between the trend to form intermetallic compounds and the specificity is unclear due to the lack of experimental data of phase diagram. For example, we cannot find a reliable experimental phase diagram^22^ for Mg-Se, Mg-Hg, Ti-Se, Ti-Te, and Ti-Ga. Those couples appear in the lower-left parts of Fig. S3b or S4b. The intermetallic compounds should be easy to form because there are strong interactions between solid and liquid metals. If these intermetallic compounds are confirmed to exist, the above correlation can be more evident.

Summary of the calculated data for Fe, Al, Mg, and Ti

Fig. S5 summarizes the calculated results for Fe (Fig. 1a), Al (Fig. 1c), Mg (Fig. S3b), and Ti (Fig. S4b). The specificity data of LME by Rostoker et al.^10^ judged the cracking with almost no plastic deformation before fracture as embrittlement (E). The fracture mode is probably GB cracking. The results show that the embrittlement pairs are within the range of GB adsorption energies from -0.7 eV/atom to 0.5 eV/atom. Within that range, embrittlement and non-embrittlement pairs appear to be jumbled. However, looking at each solid metal individually, the closer to zero energy, the more prominent the embrittlement pairs are. In addition, if this near-zero energy condition (weak interaction criterion) is not a sufficient condition but a necessary condition, it is understandable that non-embrittlement pairs sometimes appear if other factors suppress embrittlement.

LME of nickel (Ni)

Although data on nickel were not available in Rostoker et al.’s experiment^10^, other detailed experimental observations were available. For this reason, we attempt to compare our calculated result of nickel with these experiments.

Luo et al. found that bismuth penetrated the grain boundaries of nickel to form a bilayer interfacial phase after annealing at 700 °C or 1100 °C^7^. These changes in grain boundary structure can be one of the causes of LME. In this case, the origin of embrittlement is semi-obvious, like gallium embrittlement of polycrystalline aluminum because the strength of grain boundaries is modified. The Ni-Bi couple also satisfies the near-zero energy condition (weak interaction criterion), as seen in Fig. S6. This condition should be satisfied for the penetration of bismuth atoms along nickel grain boundaries.

Lynch investigated liquid metal embrittlement by potassium, sodium, mercury, and lithium on polycrystalline nickel^29^. The degree of embrittlement of polycrystalline nickel is most substantial in lithium, followed by mercury and sodium. The embrittlement by sodium is weak, and that by potassium does not occur. Lithium is closest to zero energy, and mercury, sodium, and potassium move away in this order, as shown in Fig. S6. In other words, the embrittlement is more significant for the elements close to zero energy, which supports the near-zero energy (weak interaction) criterion.

LME of copper (Cu) and zinc (Zn)

Nicholas summarized a survey on many works of literature about LME until the 1970s^19^. Copper and zinc were intensively studied. There are well-known embrittling couples like Cu-Sn, Cu-Pb, Cu-Bi, Zn-Ga, Zn-Sn, and Zn-Hg. However, there is no systematic study for the specificity of LME, which uses several or more liquid metal elements under the same test conditions for high-strength-type solid metal alloys of copper and zinc. Therefore, we cannot make a more detailed comparison.

For copper, the GB adsorption energy of all liquid metals appears within a narrow range from -0.6 to 0.6 eV/atom around zero energy, as shown in Fig. S7. It indicates that most liquid metals may satisfy the near-zero energy condition (weak interaction criterion). Depending on the experimental works in literature, some liquid metals such as mercury, indium, and gallium belong to the embrittlement group^19^.

For zinc, the calculations are conducted only for lattice dissolution energy, as shown in Fig. S8, because its high-index GB and surface structures are too unstable to calculate the adsorption energy of liquid metal atoms. We used the 4x4x3 hexagonal superlattice (96 atoms/cell) with the cutoff of 360 eV and 4x4x5 k-point mesh.

Relationship between LME and intermetallics formation

We have also found another exciting trend in Fig. 1ac. We marked and divided liquid metal elements into two groups: (e) binary intermetallic compound exists with solid metal, (n) does not exist, or unknown^22^. In the upper-right part, intermetallic compounds do not exist or are unknown to exist. In the lower-left part, where energetically stable, intermetallic compounds exist. The alternative judgment of existing or not-existing intermetallic compounds cannot be a criterion in the specificity of LME, which is well known^32^. However, a continuous view from the calculated energies, as shown in Fig. 1ac, indicates that the embrittlement seems to occur in a transition region from where the compounds exist to where they do not exist. We can see the same trend in nickel (Fig. S6b). The same trend is not evident for magnesium, titanium, copper, and zinc (Fig. S3b, S4b, S7b, and S8b), probably because of the lack of data.

LME due to corrosion

In the last part of the main text, we mentioned type (c) LME according to the classification^11^ by Norkett et al. In this type, the corrosion dissolution reaction proceeds selectively along grain boundaries. For example, the preferential dissolution of chromium and nickel from stainless steel in molten sodium was reported^30^. However, we have not yet investigated the correlation between this type of LME and the near-zero energy concept (weak interaction criterion).

Microcrack growth assisted by surface adsorption

Rice and Wang have shown that the cohesive energy of iron GB affected by solute segregation governs the ductile-brittle transition temperature (DBTT) of GB embrittlement^12^, which is well known as the Rice-Wang theory. Surface adsorption has the effect of reducing the cohesive energy of GB. In the case of LME and hydrogen embrittlement, where the embrittling elemental atoms can move during fracture, the reduction of cohesive energy is considered to be enhanced.

First-principles calculations have supported this theory by calculating iron GB cohesive energy affected by solute (B, C, P, and S) segregation compared with the experimental DBTT data of high-purity iron^13^.

The Rice-Wang theory assumes that bond-breaking and dislocation emissions occur concomitantly at a microcrack tip. Based on this idea, microcrack grows when bond-breaking is superior to dislocation emissions, while microcrack blunting occurs in the opposite case. The same idea appears in the physical model of microcrack growth on temper embrittlement proposed by Jokl, Vitek, and McMahon^14^ and of low-temperature embrittlement of steel by Higashida and Tanaka^15^. These two physical models indicate that the amount of dislocation emission from the crack tip depends on the cohesive energy of the fracture path plane, like GB and crystal plane.

Yamaguchi and Kameda have shown a good correlation between the GB cohesive energy calculated from first-principles and the fracture stress and toughness in temper embrittlement of Ni-Cr steel with solute segregation (Sb, Sn, P)^16^. Their results support the above idea.

For this reason, we consider that the microcrack growth of LME also occurs when the bond-breaking is dominant rather than dislocation emission at the microcrack tip, as shown in Fig. S9. Note that we have no idea about the effect of surface adsorption on dislocation emission because the computational simulation of dislocation emission is not easy.

Relationships of material parameters and test conditions with weak interaction criterion

Although many necessary conditions for LME to occur, we believe the following two conditions are the most basic. One condition is that liquid metal atoms adsorb to the solid metal surface, resulting in energetic stabilization and thus promoting fracture surface formation. The second condition is that the near-zero energy condition (weak interaction criterion), which we have proposed in this paper, requires liquid metal atoms to penetrate at least one or several atomic distances into a solid metal lattice or grain-boundary plane to promote fracture surface formation by surface adsorption of some atoms cooperatively. As explained in Fig. 2 and Fig. S9, we consider the entry process of a liquid metal atom in a situation where the interatomic bond is about to be broken by high stress at a microcrack-tip region.

We found the surface energy lowering effect by surface adsorption for all solid-liquid metal pairs (Fig. 1bd, Fig. S3a, S4a, S6a, and S7a). Moreover, the size of the effect is considerably large for all the couples. Therefore, although the surface energy lowering effect is necessary for embrittlement, its strength may have little effect on the specificity of LME. These strong surface adsorptions indicate that the wetting effect on the surface is one of the requirements for LME. LME will not occur if some reasons inhibit wetting of the surface.

On the other hand, we believe that the energy associated with the atomistic penetration process, which is the origin of the near-zero energy condition (weak interaction criterion) we proposed, is highly dependent on the solid-liquid metal pairs, as we have calculated, and thus will significantly affect the specificity of LME. At this point, our concept is different from the analytical results using thermodynamic parameters by Kelley and Stoloff^32^.

Based on our hypothesis, the atomistic penetration process, which is the basis of the near-zero energy (weak interaction) concept, requires a specific temperature because thermal activation is required. As a ductility trough^9,19,28,32^, liquid metal embrittlement occurs within a specific temperature range. If the temperature is too high, it weakens the surface energy lowering effect due to surface adsorption, and embrittlement may not occur. On the other hand, dislocation nucleation and movement become active at the microcrack-tip region at high temperatures, and the crack-tip blunting becomes significant to suppress the embrittlement. At higher temperatures, the penetration of liquid metal atoms into the grains takes precedence over the grain boundaries, resulting in the reduction of liquid atoms at the grain boundaries and the suppression of grain boundary fracture. An example is the Al-Ga couple^7^.

Along with temperature, strain rate and exposure time affect the LME. Although details are unknown, it is reasonable to consider that atomic-level penetration of liquid metal atoms at the microcrack tip takes some time, and therefore embrittlement may not occur if the strain rate is high or the exposure time is insufficient. Oppositely, brittleness can be more severe when the strain rate is higher in some cases. As in the case of Al-Ga, when the intragranular penetration of Ga becomes significant, grain boundary cracking is suppressed, and this may be due to the loss of Ga at grain boundaries. The susceptibility to cracking at grain boundaries also depends on the grain size and microstructure. Thus, pre-strain also affects LME. Since liquid metal atoms in grains and grain boundaries affect LME, various material parameters mutually depend in a complex manner.

The strength of the material strongly affects the extent of the ductility trough. The higher the material strength, the higher the stress concentration at the microcrack-tip region so that the liquid metal atoms are more likely to penetrate the lattice plane or grain-boundary plane of the solid metal. Moreover, blunting of the crack tip is suppressed by decreasing dislocation activity. Due to these effects, the range of ductility troughs may expand as the material strength increases. These behaviors can be the reasons why high-strength commercial alloys easily exhibit LME rather than low-strength pure metals.

Among the embrittlement couples in LME, the polycrystalline Al and liquid Ga pair may be extreme where embrittlement occurs without applied stress^2^. In this case, the Ga atoms penetrate the Al grain-boundary atomic planes, and the fracture surfaces form without applied stress. Furthermore, our calculated results indicate that the adsorption energy of the Ga atom on the Al grain boundary shows a small negative value, as shown in Fig. 1c. It may suggest that the Ga atoms can penetrate without stress depending on the conditions.

There is a rough correlation between the electronegativity difference between solid and liquid metals and the degree of embrittlement^31,32^. LME is less likely to occur if the electronegativity difference is significant. This general trend may fit well with our near-zero energy (weak interaction) concept. The more significant the difference in electronegativity, the less metallic bonding is likely to form because a weak ionic bonding becomes dominant. It indicates that liquid metal atoms entering solid metal will be unstable, not satisfying the near-zero energy condition.

Fortunately, it is easy to understand the correlation between the electronegativity and the degree of embrittlement of liquid metal elements added to mercury, as observed in the mercury embrittlement of aluminum^32^. In Fig. 1c, Hg is more energetically unstable than Ga, and the electronegativity of Hg is far from Al than Ga. This behavior obeys the above explanation. However, the electronegativity difference is only one of the indicators for the ionicity of chemical bonding. This difference alone cannot indicate the interaction of liquid metal elements when they enter solid metals. For this reason, the correlation of electronegativity with LME may be limited.

As Norkett et al.^11^ pointed out in their recent review, most solid-liquid metal couples that do not give rise to LME were often determined to be non-embrittlement based on only a few test conditions and therefore need to be confirmed by more detailed experiments. We agree with the view by Norkett et al. that many of the pairs identified as non-embrittlement couples will be the embrittlement ones by careful experiment under certain conditions. Our study indicates that all solid-liquid couples we investigated can potentially cause LME because they all have the surface energy lowering effect. Therefore, the nonattainment of the near-zero energy condition (weak interaction criterion) prevents LME for some couples, depending on experimental conditions.

**References:**

1. A. Legris, G. Nicaise, J.-B. Vogt, J. Foct, Liquid metal embrittlement of the martensitic steel 91: influence of the chemical composition of the liquid metal. Experiments and electronic structure calculations. *J. Nucl. Mater.* ***301***, 70-76 (2002). doi:10.1016/S0022-3115(01)00730-9
2. M. G. Nicholas and C. F. Old, Liquid metal embrittlement. *J. Mater. Sci.* ***14***, 1-18 (1979). doi:10.1007/BF01028323
3. S. P. Lynch, A fractographic study of hydrogen-assisted cracking and liquid-metal embrittlement in nickel. *J. Mater. Sci.* ***21***, 692-704 (1986). doi:10.1007/BF01145543
4. R. H. Hiltz, The corrosion of stainless steel in oxygen-contaminated sodium at 1200 F and 1400 F. in J. E. Draley and J. R. Weeks, Ed., *Corrosion by liquid metals* (Springer US, Boston, MA 1970). doi:10.1007/978-1-4684-1845-3_5
5. M. J. Kelley, N. S. Stoloff, Analysis of liquid metal embrittlement from a bond energy viewpoint, *Metallur. Trans.* ***6A***, 159-166 (1975).
   doi:10.1007/BF02673684
6. B. Joseph, M. Picat, F. Barbier, Liquid metal embrittlement: a state-of-the-art appraisal. *Eur. Phys. J. AP* ***5***, 19-31 (1999). doi:10.1051/epjap:1999108
7. K. Momma, F. Izumi, VESTA 3 for three-dimensional visualization of crystal, volumetric and morphology data. *J. Appl. Crystallogr.* ***44***, 1272-1276 (2011). doi:10.1107/S0021889811038970

Table S1. Data for solid elements and parameters of cell modeling. GB: grain boundary. TB: twin boundary. Angles are shown except for 90°.

|  | Fe | Al | Ni | Cu | Mg | Ti |
| --- | --- | --- | --- | --- | --- | --- |
| Basic cell | BCC | FCC | FCC | FCC | HCP | HCP |
| a (Å) | 2.83 | 4.04 | 3.52 | 3.63 | 3.23 | 2.95 |
| c (Å) | - | - | - |  | 5.27 | 4.68 |
| c/a | - | - | - |  | 1.63 | 1.59 |
| magnetism | FM | NM | FM | NM | NM | NM |
| Supercell size | 5x5x5 | 4x4x4 | 4x4x4 | 4x4x4 | 5x5x3 | 5x5x3 |
| Gamma-centered k-point mesh | 4x4x4 | 4x4x4 | 4x4x4 | 4x4x4 | 4x4x4 | 4x4x4 |
|  |  |  |  |  |  |  |
| GB(TB) cell |  |  |  |  |  |  |
| **a** // | [1 -1 0] | [1 0 0] | [1 0 0] | [1 0 0] | [1 -1 0]* | [1 -1 0]* |
| length (Å) | 8.01 | 8.08 | 7.04 | 7.25 | 11.19 | 10.22 |
| **b** // | [1 1 -2] | [0 2 -1] | [0 2 -1] | [0 2 -1] | [3 3 -2]* | [1 -2 -1]* |
| length (Å) | 6.94 | 9/03 | 7.87 | 8.11 | 14.32 | 13.86 |
| **c** // | [1 1 1] | [0 1 2] | [0 1 2] | [0 1 2] | [16 16 9]* | [32 32 19]* |
| length (Å) | 39.24 | 40.13 | 31.49 | 32.46 | 50.17 | 49.74 |
| α, β, γ |  |  |  |  |  | α=89.95° ,  γ=111.63° |
| Grain boundary | Σ3(1 1 1) | Σ5(0 1 2) | Σ5(0 1 2) | Σ5(0 1 2) | TB(1 1 -2 3) | TB(1 1 -2 3) |
| Surface | (1 1 1) | (0 1 2) | (0 1 2) | (0 1 2) | (1 1 -2 3) | (1 1 -2 3) |
| Number of atoms | 100 | 108 | 84 | 84 | 196 | 212 |
| Gamma-centered k-point mesh | 5x5x1 | 5x5x1 | 5x5x1 | 5x5x1 | 4x4x1 | 4x4x1 |

*) three-index scheme for hcp structure

BCC: body-centered cubic

FCC: face-centered cubic

HCP: hexagonal

FM: ferromagnetic

NM: non-magnetic

Table S2. Data for liquid elements. *T*_m_: melting temperature.

| Liquid metal (X) | *T*_m_ [℃] | Crystal structure | Latent heat (enthalpy) of fusion [eV/atom] at *T*_m_ (*16*) |
| --- | --- | --- | --- |
| Hg | -39 | RHL | 0.02 |
| Ga | 30 | ORC | 0.06 |
| Na | 98 | BCC | 0.03 |
| In | 157 | TET | 0.03 |
| Li | 181 | BCC | 0.03 |
| Se | 220 | HEX | 0.06 |
| Sn | 232 | TET | 0.07 |
| Bi | 271 | RHL | 0.11 |
| Tl | 304 | HEX | 0.04 |
| Cd | 321 | HEX | 0.07 |
| Pb | 328 | FCC | 0.05 |
| Zn | 420 | HEX | 0.08 |
| Te | 450 | HEX | 0.18 |
|  |  |  |  |
| Cs | 29 | BCC | 0.02 |
| Rb | 39 | BCC | 0.02 |
| K | 64 | BCC | 0.02 |

FCC: Face-Centered Cubic

BCC: Body-Centered Cubic

TET: Tetragonal

ORC: Orthorhombic

HEX: Hexagonal

RHL: Rhombohedral

Table S3. Calculated grain-boundary(GB) or twin-boundary (TB), and its fracture surface energies.

|  | Fe | Al | Ni | Cu | Mg | Ti |
| --- | --- | --- | --- | --- | --- | --- |
|  | Σ3(111) | Σ5(012) | Σ5(012) | Σ5(012) | TB(11-23) | TB(11-23) |
| GB or TB energy  (J/m^2^) | 1.52 | 0.50 | 1.29 | 0.87 | 0.38 | 0.60 |
| Fracture surface energy  (J/m^2^) | 2.69 | 0.98 | 2.38 | 1.60 | 0.75 | 2.00 |

| a | b | c | d | e |
| --- | --- | --- | --- | --- |
| 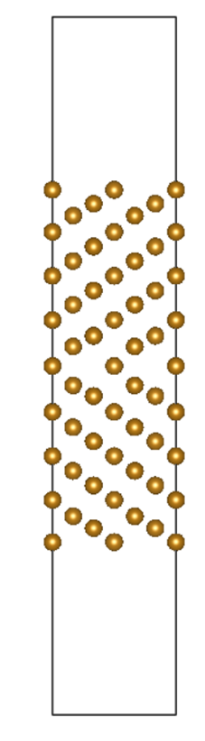  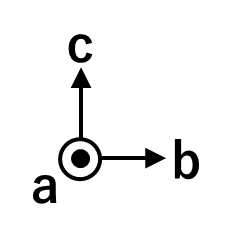 | 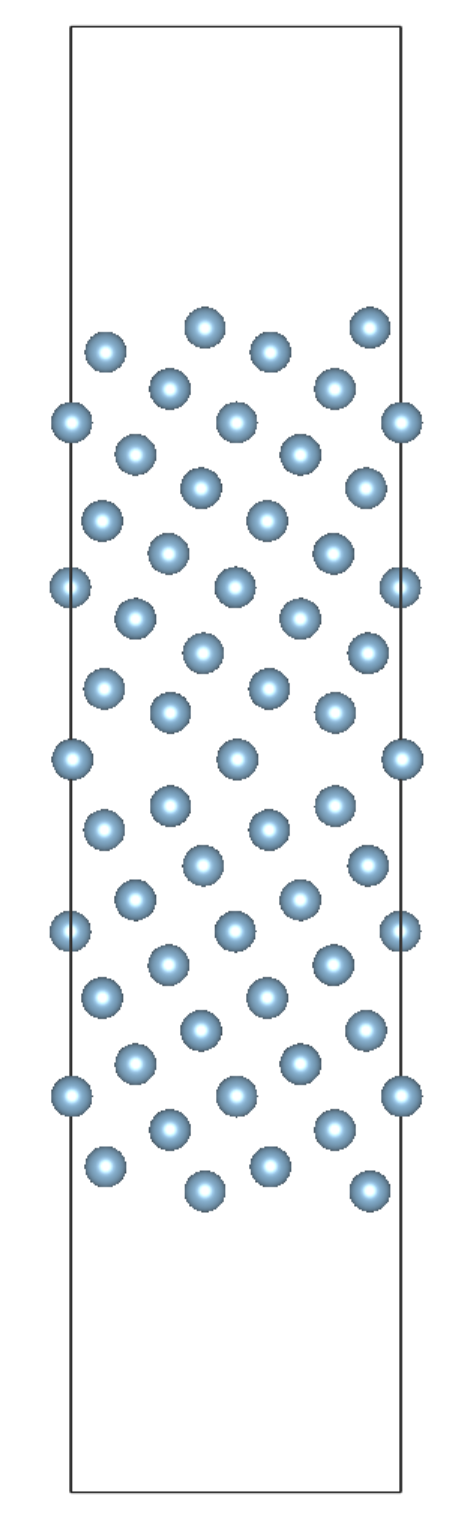  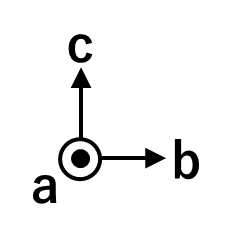 | 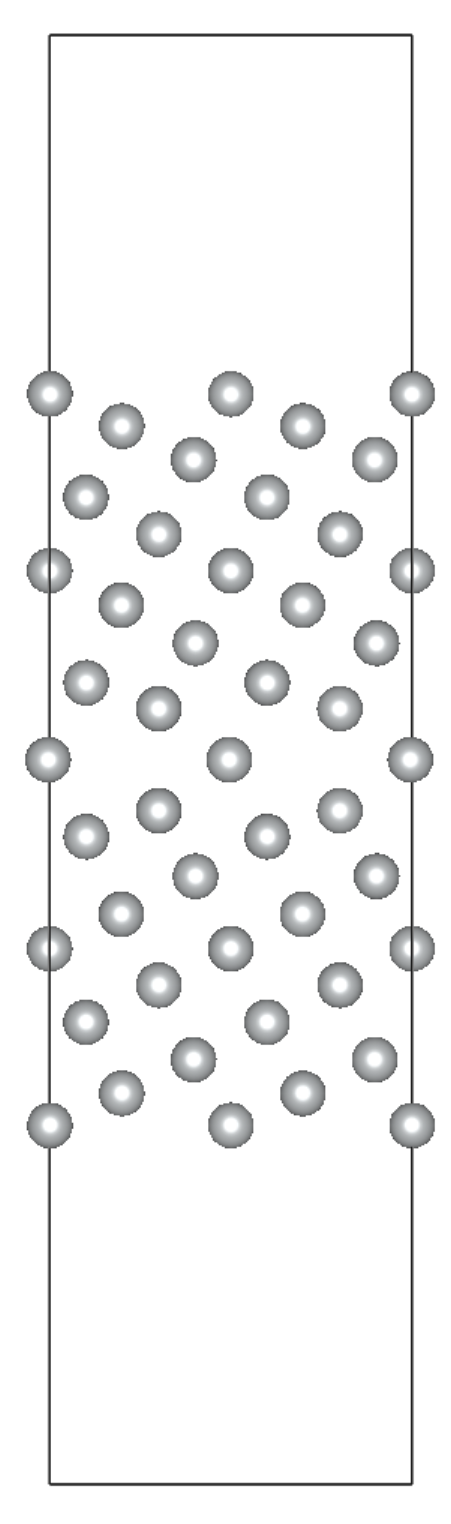  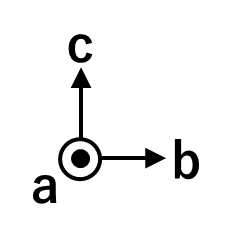 | 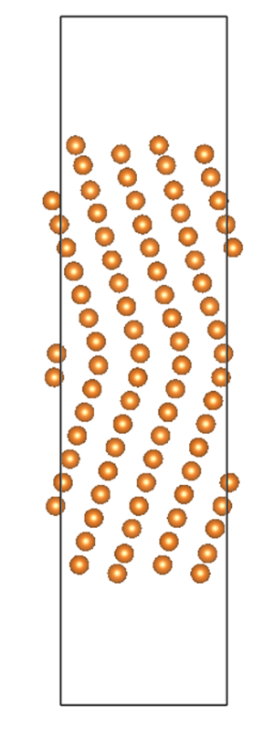  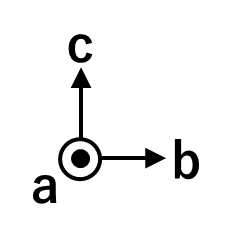 | 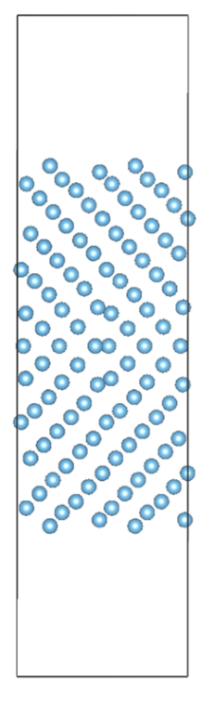  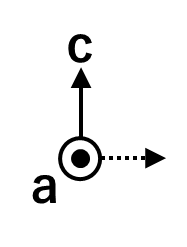 |

Fig. S1. Unit cell modeling for GB or twin boundary (TB) and fracture surfaces for solid metals. a, Σ3(111) GB of body-centered cubic iron (Fe). b, Σ5(012) GB of face-centered-cubic aluminum (Al). c, Σ5(012) GB of face-centered-cubic nickel (Ni, Cu). d, TB(11-23) of hexagonal magnesium (Mg). e, TB(11-23) of hexagonal titanium (Ti). γ = 111.63°. Cell parameters are listed in table S2. Structures were visualized by VESTA software^33^.


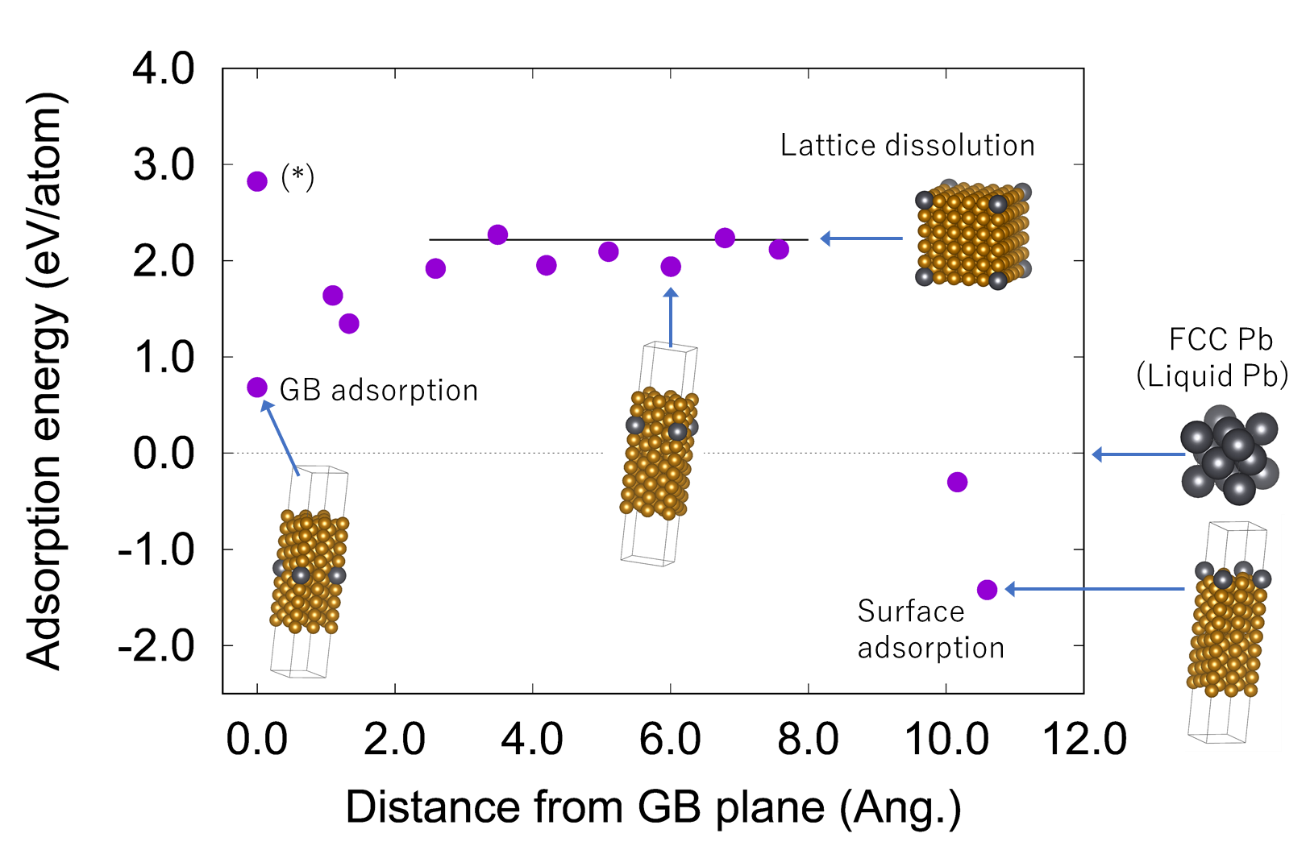


Fig. S2. The relationship in the calculated energies of a Pb atom, for example. Inserted figures indicate the position of a Pb atom in the cell for each state. (*) The vacant site on the GB plane is occupied by a Pb atom, which is unstable energetically.

| a | 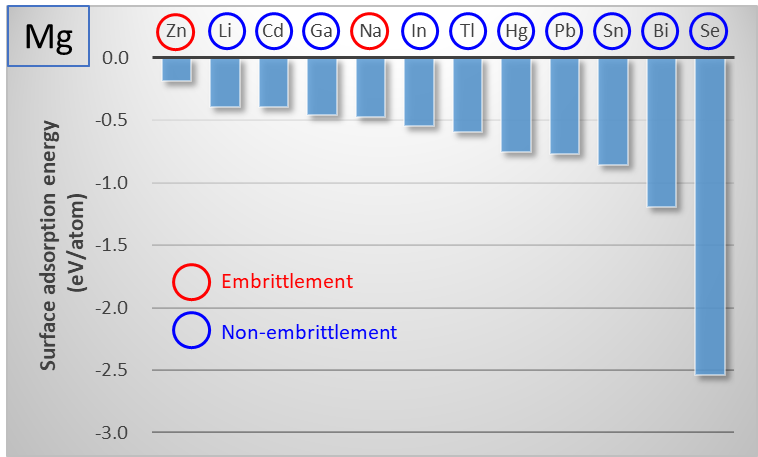 |
| --- | --- |
| b | 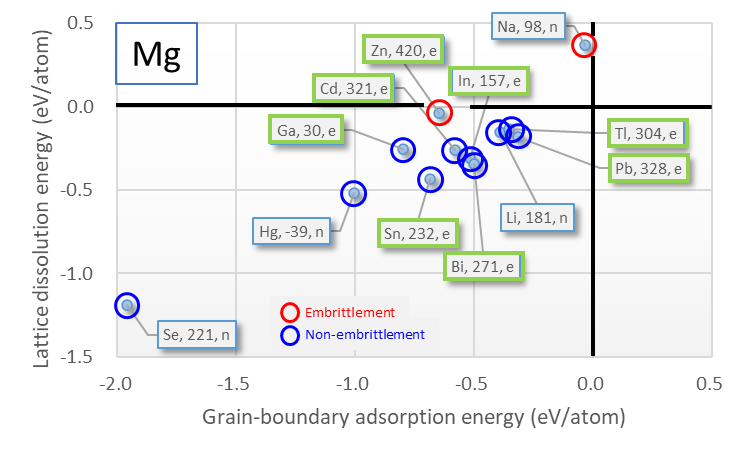 |

Fig. S3. Calculated results for liquid metal elements in solid Mg. a, The surface adsorption energy of liquid metal elements on the Al (012) surface. b, Dissolution energy in the crystal lattice and adsorption energy at Σ5(012) grain boundary of liquid metal elements. The red circle indicates embrittlement and the blue one non-embrittlement in Rostoker’s surveillance test^1^. The green box indicates that a binary intermetallic compound with Mg exists (e), and the other box does not (n). Reliable experimental data of binary phase diagrams with Mg are unavailable for Na, Hg, and Se^22^.

| a | 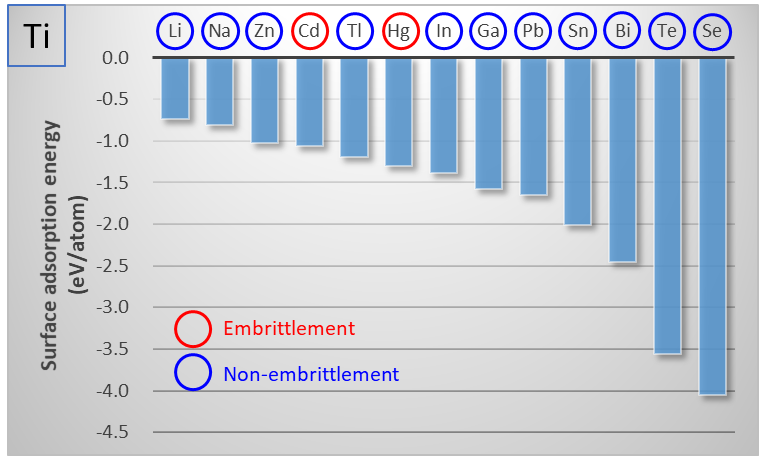 |
| --- | --- |
| b | 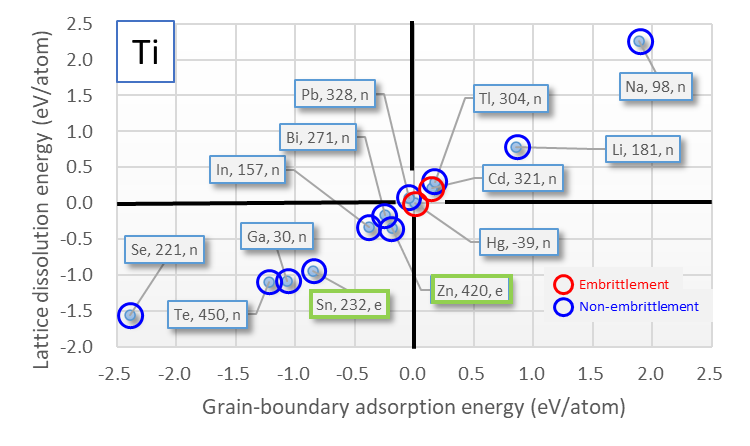 |

Fig. S4. Calculated results for liquid metal elements in solid Ti. a, The surface adsorption energy of liquid metal elements on the Al (012) surface. b, Dissolution energy in the crystal lattice and adsorption energy at Σ5(012) grain boundary of liquid metal elements. The red circle indicates embrittlement and the blue one non-embrittlement in Rostoker’s surveillance test^1^. Note that the embrittling elements are Cd and Hg. The green box indicates that a binary intermetallic compound with Ti exists (e), and the other box does not (n). Reliable experimental data of binary phase diagrams with Ti are available only for Sn and Zn^22^.

| 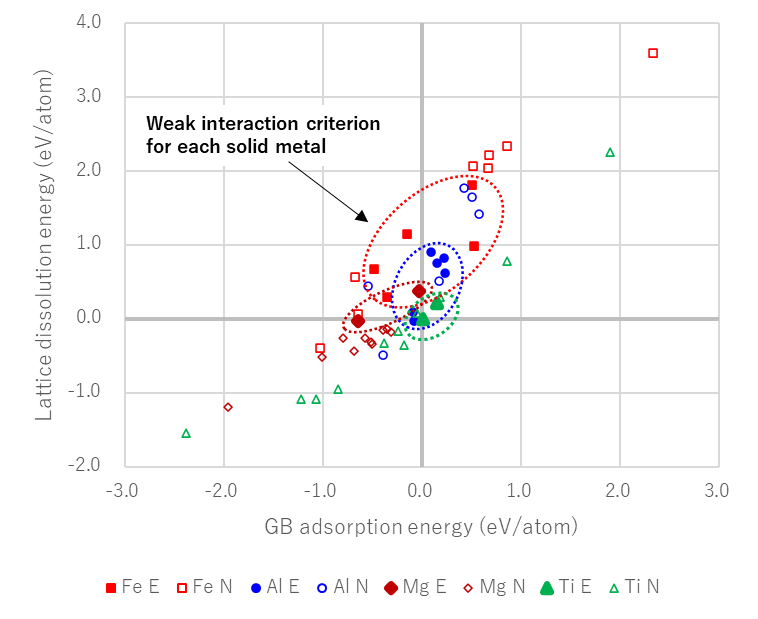 |
| --- |

Fig. S5. Summary of calculated results for Fe, Al, Mg, and Ti compared with the specificity data of LME. The specificity data are taken from Rostoker et al.^10^ E: Embrittlement. N: Non-embrittlement. The symbols of “Mg E” and “Ti E” are enlarged to make them easier to see.

| a | 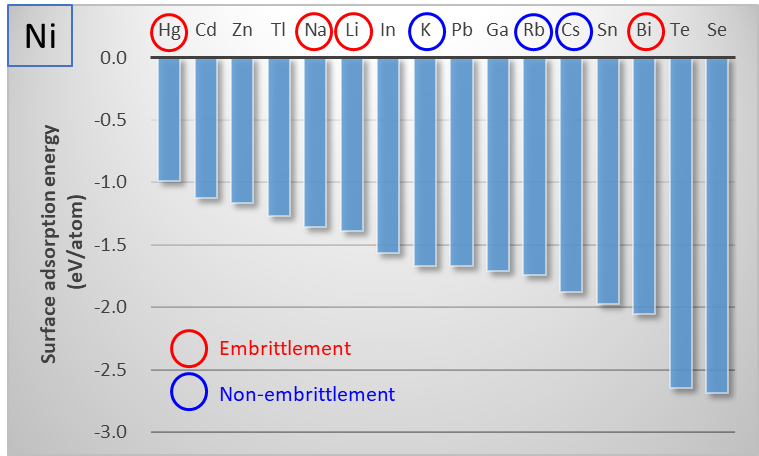 |
| --- | --- |
| b | 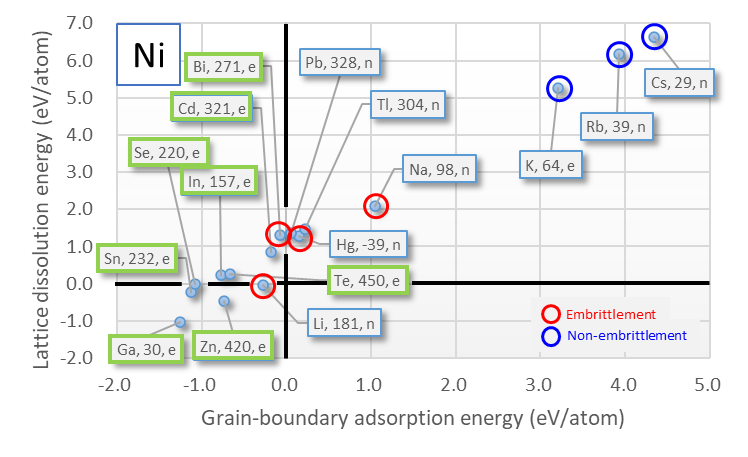 |

Fig. S6. Calculated results for liquid metal elements in solid Ni. a, The surface adsorption energy of liquid metal elements on Ni (012) surface. b, Dissolution energy in the crystal lattice and adsorption energy at Σ5(012) grain boundary of liquid metal elements. The red circle indicates embrittlement and the blue one non-embrittlement in Lynch’s surveillance test^30^ and Luo et al.’s result of polycrystalline Ni embrittled by Bi^5^. The green box indicates that a binary intermetallic compound with Ni exists (e), and the other box does not (n). Note that the embrittling elements are Li, Bi, Hg, and Na. Reliable experimental data of binary phase diagrams with Ni are unavailable for Cs, Rb, K, Na, Tl, and Li^22^.

| a | 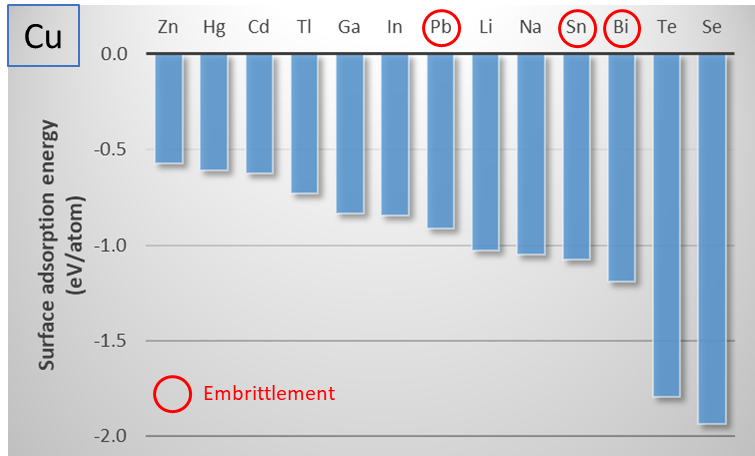 |
| --- | --- |
| b | 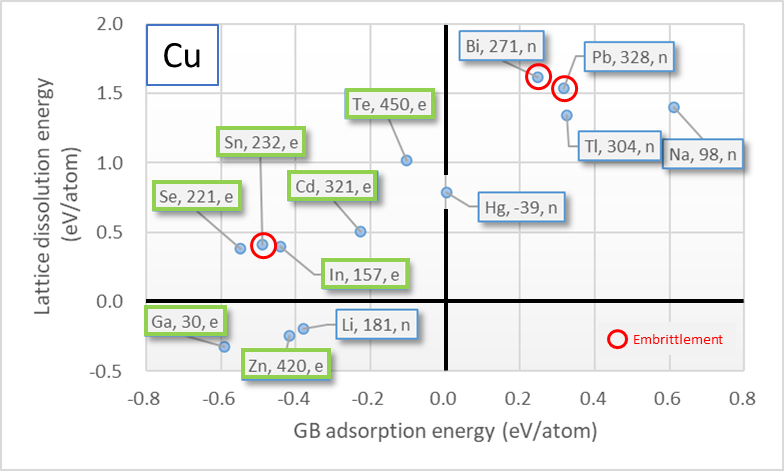 |

Fig. S7. Calculated results for liquid metal elements in solid Cu. a, The surface adsorption energy of liquid metal elements on Cu (012) surface. b, Dissolution energy in the crystal lattice and adsorption energy at Σ5(012) grain boundary of liquid metal elements. Note that the well-known embrittling elements are Sn, Pb, and Bi, but there is no systematic experiment investigating the specificity of LME. The green box indicates that a binary intermetallic compound with Cu exists (e), and the other does not (n).


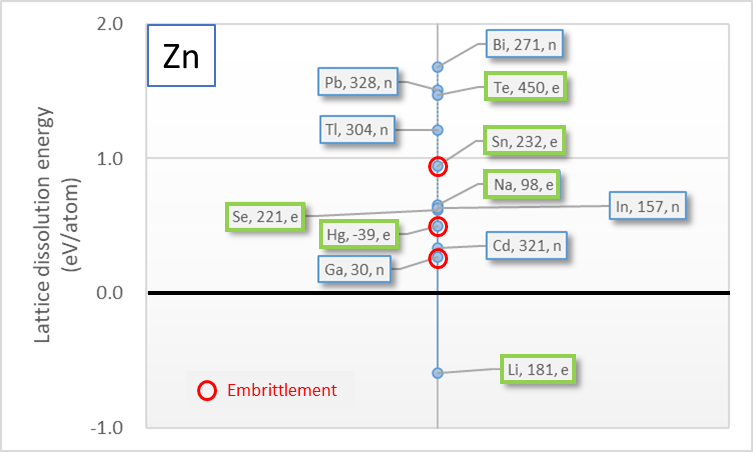


Fig. S8. Calculated results for liquid metal elements in solid Zn. Only lattice dissolution energies in the hcp crystal lattice are shown. The red circle indicates embrittlement in Nicholas’s literature survey^19^. Note that the well-known embrittling elements are Hg, Ga, and Sn. However, there is no systematic experiment for specificity. The green box indicates that a binary intermetallic compound with Zn exists (e), and the other box does not (n).

| **a** | 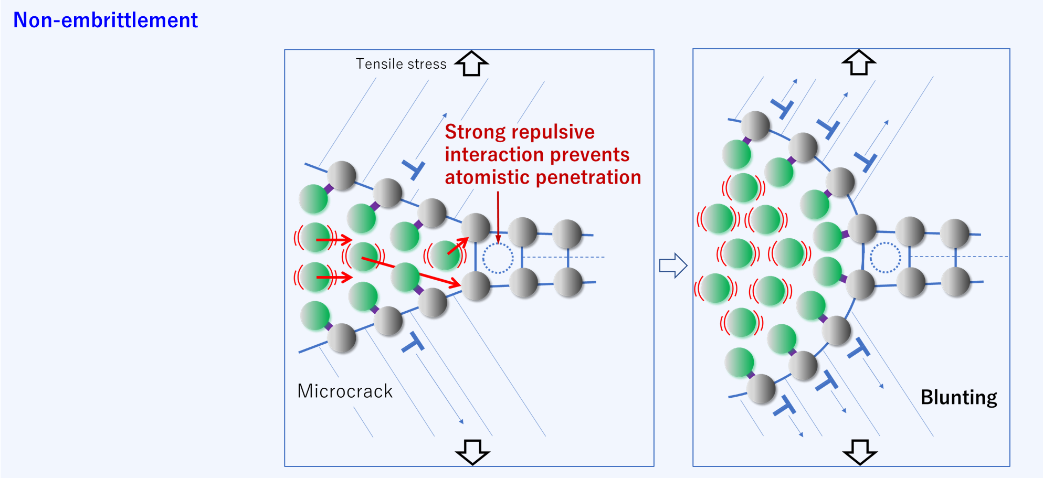 |
| --- | --- |
| **b** | 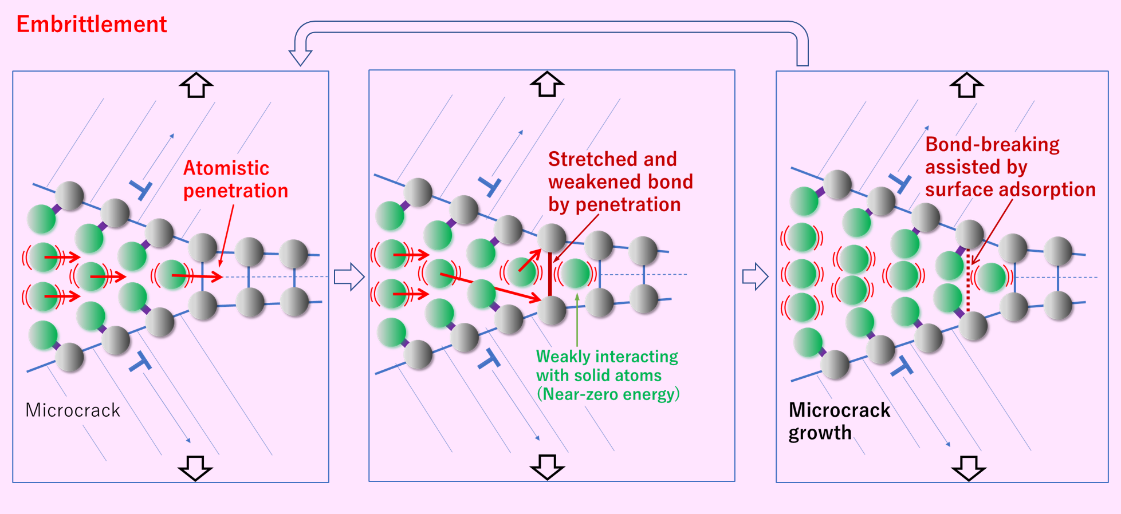 |
| **c** | 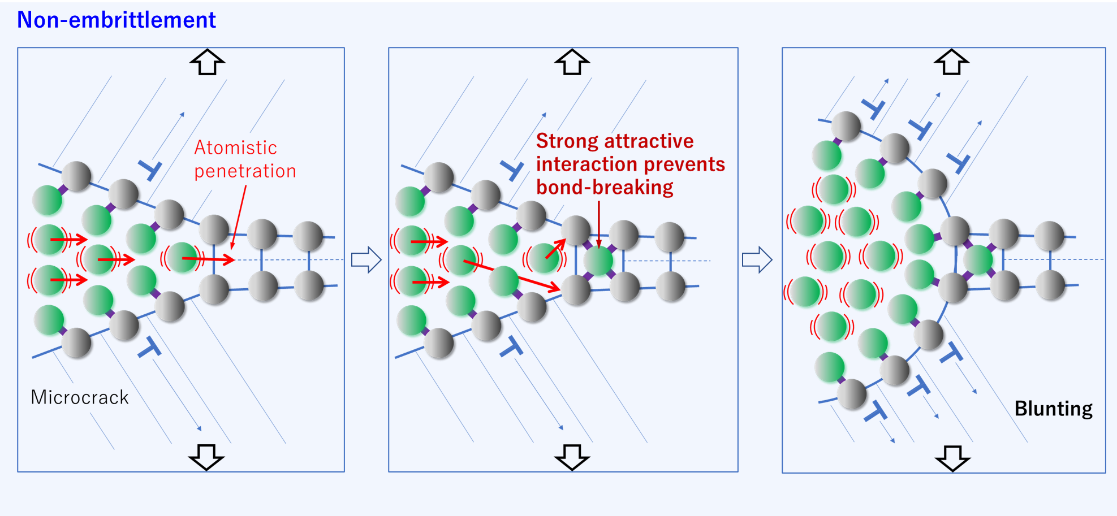 |

**Fig. S9. A proposed atomistic process of embrittlement and non-embrittlement. a**, Because a liquid metal atom intensely and repulsively interacts with the surrounded solid atoms, a liquid atom cannot penetrate GB, lath boundary, or crystal lattice plane. Thus, the bond-breaking does not occur, resulting in microcrack blunting. **b**, For the sake of appropriate weak interaction (near-zero energy) between solid and liquid atoms, a liquid metal atom penetrates solid’s GB and induces bond-breaking assisted by surface adsorption, resulting in microcrack growth. **c**, Because a liquid metal atom intensely and attractively interacts with the surrounded solid atoms, a liquid atom can penetrate GB. However, the subsequent bond-breaking is suppressed, resulting in microcrack blunting. We assume that the dislocation emission occurs concomitantly with bond-breaking and is not affected by surface adsorption. See Fig. 3.


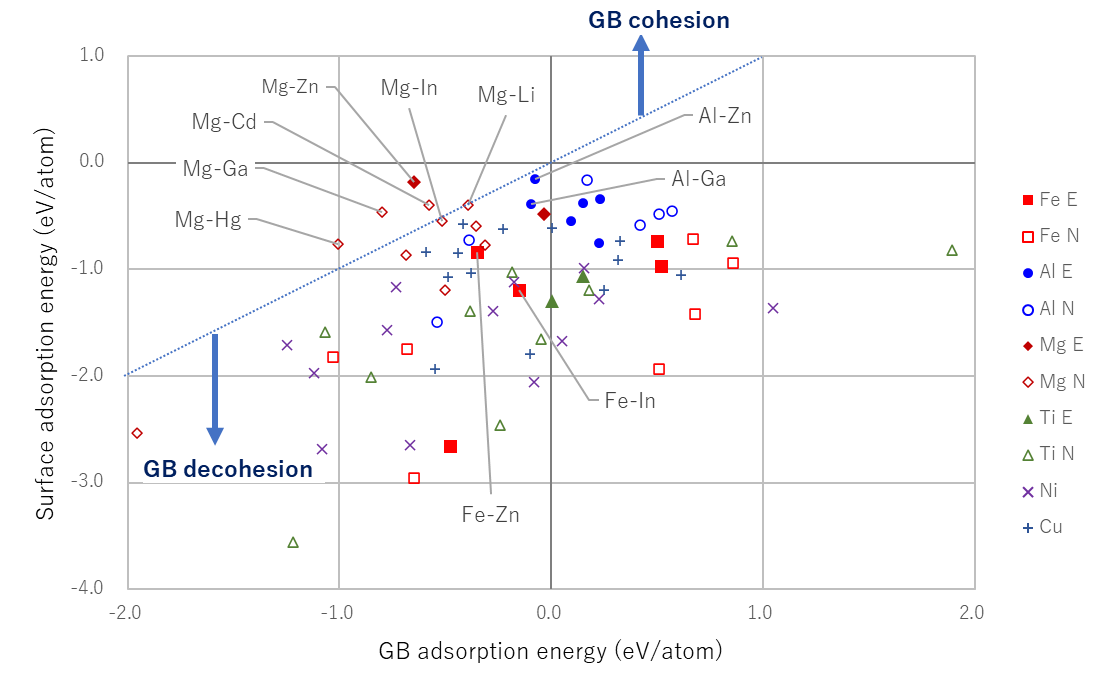


**Fig. S10. GB cohesion/decohesion effect of liquid metal atoms when they penetrate and diffuse into solid GB.** The strength of decohesion is measured by the difference between surface and GB adsorition energies. Almost all couples exhibit the GB decohesion effect, realized when surface adsorption is stronger than GB adsorption. Some exceptions are found only in Mg-X couples (X=Hg, Ga, Cd, Zn). The data symbols are the same as in Fig. S5.
